# Butyrate dictates ferroptosis sensitivity through FFAR2-mTOR signaling

**DOI:** 10.1038/s41419-023-05778-0

**Published:** 2023-04-25

**Authors:** GuoYan Wang, SenLin Qin, Lei Chen, HuiJun Geng, YiNing Zheng, Chao Xia, JunHu Yao, Lu Deng

**Affiliations:** 1 College of Animal Science and Technology, Northwest A&F University, Yangling, Shaanxi 712100 China; 2 Division of Laboratory Safety and Services, Northwest A&F University, Yangling, Shaanxi 712100 China

**Keywords:** Cell signalling, Cell death

## Abstract

Evidence shows that short-chain fatty acids (SCFAs) play an important role in health maintenance and disease development. In particular, butyrate is known to induce apoptosis and autophagy. However, it remains largely unclear whether butyrate can regulate cell ferroptosis, and the mechanism by which has not been studied. In this study, we found that RAS-selective lethal compound 3 (RSL3)- and erastin-induced cell ferroptosis were enhanced by sodium butyrate (NaB). With regard to the underlying mechanism, our results showed that NaB promoted ferroptosis by inducing lipid ROS production via downregulating the expression of solute carrier family 7 member 11 (SLC7A11) and glutathione peroxidase 4 (GPX4). Moreover, the FFAR2-AKT-NRF2 axis and FFAR2-mTORC1 axis accounts for the NaB-mediated downregulation of SLC7A11 and GPX4, respectively, in a cAMP-PKA-dependent manner. Functionally, we found that NaB can inhibit tumor growth and the inhibitory effect could be eliminated by administrating MHY1485 (mTORC1 activator) and Ferr-1 (ferroptosis inhibitor). Altogether, in vivo results suggest that NaB treatment is correlated to the mTOR-dependent ferroptosis and consequent tumor growth through xenografts and colitis-associated colorectal tumorigenesis, implicating the potential clinical applications of NaB for future colorectal cancer treatments. Based on all these findings, we have proposed a regulatory mechanism via which butyrate inhibits the mTOR pathway to control ferroptosis and consequent tumorigenesis.

## Introduction

Short-chain fatty acids (SCFAs) are produced as a result of the fermentation of dietary fiber and indigestible starch in the colonic lumen by commensal bacteria and play important roles in health maintenance and disease development [1, 2]. Research has shown that acetate, propionate, and butyrate account for more than 90% of the total SCFAs [3]. Butyrate, which is generally considered to be a source of energy [4], is produced by the condensation of two acetyl-CoA molecules as substrates to form acetoacetyl-CoA, which is then reduced to butyryl-CoA [5]. There is growing evidence that butyrate plays an important role in immunity. The antimicrobial activity of butyrate is associated with the inhibition of HDAC3 activity, induction of anti-inflammatory macrophage production [6], and improvement of host defense and antimicrobial peptide production [7]. The current research on butyrate mainly focuses on immune regulation, but there is some emerging evidence that butyrate plays an important role in ferroptosis [8, 9], which is a recently recognized form of cell death. However, the mechanism by which butyrate regulates ferroptosis has not been studied yet.

Ferroptosis is a form of regulated cell death triggered by iron-dependent lipid peroxidation of the cellular membrane. It is distinctive from other forms of regulated cell death, such as apoptosis and necroptosis, and has an important role in various physical conditions and diseases, including cancers [10]. The decisive trigger for ferroptosis is the peroxidation of polyunsaturated fatty acid in the cell membrane, which is regulated by multiple enzymes [11], including acyl-coenzyme A synthetase long chain family member 4 (ACSL4), lysophosphatidylcholine acyltransferase 3 [12], and lipoxygenases (ALOXs) [13]. To counteract the adverse effects of lipid peroxidation, cells have evolved ferroptosis defense systems to suppress lipid peroxidation [11]. Studies have shown that cells take up extracellular cystine via solute carrier family 7 member 11 (SLC7A11). The extracellular cystine is subsequently reduced to two molecules of cysteine, which is used for protein and, in particular, glutathione (GSH) biosynthesis and plays a vital role in defending against ferroptosis [14]. Glutathione peroxidase 4 (GPX4), a central GSH-utilizing enzyme, utilizes GSH as its cofactor for counteracting lipoxygenase activities and phospholipid/cardiolipin oxidation events, thereby preventing cells from undergoing ferroptosis [15]. In addition, nuclear factor erythroid 2-related factor 2 (NRF2) is the master regulator of redox homeostasis systems [16] and is translocated to the nucleus, where it binds to antioxidant response elements and promotes the expression of its downstream target genes (including GSH), chemoresistance, and cytoprotection [17]. It has been shown that the stability of NRF2 is controlled by Kelch-like ECH-associated protein 1 [18], but accumulating evidence has revealed that phosphoinositide 3-kinase (PI3K)-protein kinase B (Akt) signaling regulates the homoeostasis of NRF2 by phosphorylating glycogen synthase kinase-3 (GSK3). This prevents NRF2 from being recognized by β-transducin repeat-containing protein (β-TRCP) Cul1-based E3 ubiquitin ligase complex SCF and, ultimately, inhibits its proteasomal degradation [19].

In addition to all the ferroptosis-related factors described above, recent studies have demonstrated that the mammalian target of rapamycin complex 2 (mTORC2) prevents ferroptosis by impairing the phosphorylation of AKT and GSK3β [20]. As a central regulator of growth and metabolism, mTOR acts as a negative modulator of ferroptosis, and its regulation of ferroptosis involves several mechanisms [21]. For example, Zhang et al. revealed that SLC7A11-mediated cystine uptake promotes the synthesis of GSH as well as GPX4 through the Rag-mTORC1-4EBP pathway and, ultimately, promotes ferroptosis resistance [22]. Further, a recent study showed that the PI3K-AKT-mTORC1 pathway suppresses ferroptosis by promoting SREBP1-SCD1-mediated MUFA synthesis [23]. Moreover, excessive autophagy may contribute to ferroptosis, previous research showed the mTORC1 pathway suppressed autophagy-dependent ferroptosis [24]. Although there is evidence that the mTOR pathway acts as a negative regulator of ferroptosis in cancer cells and that the regulation of ferroptosis by mTOR involves several mechanisms, so far, it is not clear whether butyrate is involved in the prevention of ferroptosis via the regulation of the mTOR pathway.

In the current study, we have tried to fill in the research gaps mentioned above by investigating the potential role of butyrate in ferroptosis and tumor growth and the underlying mechanism of mTOR activation in response to butyrate.

## Materials and methods

### Plasmids, antibodies, and reagents

Flag-SLC7A11, Flag-βTRCP1, Flag-GSK3β, and Myr-AKT were provided by P. Wang (Tongji University, Shanghai, China). The anti-Flag (F7425), Erastin (E7781), ferrostatin-1 (ferr-1, SML0583), deferoxamine (DFO, D9533), RSL3 (SML2234), Insulin (I0310000), Torin1 (475991), Cycloheximide (CHX), N-acetylcysteine (NAC, A7250), and secondary antibodies were obtained from Sigma-Aldrich (MO, USA). H89 2HCl (H89, S1582), MHY1485 (S7811), and bafilomycin A1 (S1413) were obtained from Selleck Chemicals (Shanghai, China). Forskolin (HY-15371) was purchased from MedChemExpress. Sodium acetate (NaAC), Sodium propionate (NaP), and Sodium butyrate (NaB) were obtained from Sangon (Shanghai, China). The antibodies against pT389-S6K (9234S/L), p-S6 (4858S), S6K (9202S), S6 (2217S), SLC7A11 (12691), pS473-AKT (9271), AKT (9272), and p-4EBP1 (9451) were obtained from Cell Signaling Technology (MA, USA). pS9-GSK3β (ab75814), GSK3β (ab32391) and Lithium chloride (LiCl, ab120853) were obtained from Abcam (Cambridge, UK). Actin (20536-1-AP), GPX4 (67763-1-Ig), p62 (18420-1-AP), LC3 (18725-1-AP) and NRF2 (16396-1-AP) were obtained from Proteintech (Chicago, USA). Amino acids (AA, 50X), β-mercaptoethanol, penicillin and streptomycin were purchased from Gibco (Grand Island, NY, USA). DMEM (amino acid-free and cystine-free) were purchased from Genetimes Technology (Shanghai, China), PBS and trypsin were purchased from HyClone (UT, USA).

### Cell culture

HCT116, HEK293T, HT29, MC38, and Caco2 cells were purchased from National Science & Technology Infrastructure (NSTI, Shanghai, China) and cultured according to the protocol. The HCT116, HEK293T, and Caco2 cells were cultured in DMEM (Hyclone, USA) and the MC38, HT29 cells were cultured in RPMI-1640 medium (Hyclone, USA) with 10% fetal bovine serum (Gibco, Grand Island, NY, USA) according to the ATCC guidelines.

### siRNA knockdown

Non-specific control siRNA and siRNAs for βTRCP1, GSK3β, and FFAR2 were purchased from GenePharma (Shanghai, China). Cells were transfected with siRNA oligonucleotides using Lipofectamine 2000 (Invitrogen, CA, USA). siRNA transfection of cells was performed according to the manufacturer’s instructions. The following siRNAs were used:

si NC: 5′-UUCUCCGAACGUGUCACGU-3′,

si β-TRCP1-1#: 5′-ACTTGCCCAGGACCCATTAAA-3′,

si β-TRCP1-2#: 5′-GCGTTGTATTCGATTTGATAA-3′,

si GSK3β-1#: 5′-GATGAATTACGGGACCCAAAT-3′,

si GSK3β-2#: 5′-CCCAAACTACACAGAATTTAA-3′,

si FFAR2-1#: 5′-GTGAAGAAGACGAGTTCTATT-3′,

si FFAR2-2#: 5′-CATCGTGATCATCGTTCAATA-3′.

### Amino acid, serum starvation and re-stimulation

Cells were rinsed with PBS and incubated in amino acid and serum-free DMEM for 6 h, and then stimulated with amino acids and insulin for the indicated time.

### Cysteine uptake assay

HT29 or HCT116 cells were treated with NaB for 6 h. To measure cysteine uptake levels of cells upon treatment, the relative intracellular cysteine concentration was assessed using Cysteine Assay Kit (Sigma, MAK255) according to the manufacturer’s instructions. All samples were normalized to cell number and conducted with three independent replicates.

### Co-immunoprecipitation (Co-IP) and western blot (WB)

Co-IP and WB were performed as previously described [25]. Transfected HEK293T cells were lysed in CHAPS lysis buffer. After sonication for 10 min, the soluble fraction of the cell lysates was isolated via centrifugation at 12 000 rpm in a microcentrifuge for 15 min at 4 °C. For Co-IP, the cell lysates were centrifuged to remove the cell debris and then were incubated in anti-Flag M2 beads for 2–3 h. The beads were boiled after extensive washing, resolved via SDS-PAGE and transferred onto nitrocellulose (NC) membrane (0.45 μm, GE). Then the membranes were probed with the primary and secondary antibodies. After being incubated with primary antibody overnight, the membranes were incubated with horseradish peroxidase (HRP)-conjugated secondary antibody at room temperature for 1 h. Finally, the proteins were detected using the imaging System (Bio-Rad, Hercules, CA, USA). ImageJ software was used to quantify the protein abundance.

### Cell viability assay

To measure cell viability, 10,000 cells per well were seeded in 96-well plates and incubated with complete DMEM or RPMI-1640 medium containing different concentrations of reagents for the indicated time, after which the medium in each well was replaced with 100 µL fresh medium containing 10% Cell Counting Kit-8 (CCK-8, K009, ZETA LIFE, CA, USA) reagent and incubation for 3 h at 37 °C, then the plate was read by a Synergy HT microplate reader (Bio-Tek, USA) at an absorbance of 450 nm.

### Quantitative real-time polymerase chain reaction (qRT-PCR) analyses

Total RNA was isolated from cells using TRIzol reagent (DP424, TIANGEN) according to the manufacturer’s instructions. The RNA samples were treated with RNase-free DNase and subjected to reverse transcription with HiScript II 1st Strand cDNA Synthesis Kit (R212-02, Vazyme). The NanoDrop 2000C Spectrophotometer (Thermo Fisher Scientific, USA) was used to detect RNA purity and concentration of each sample. qRT-PCR analysis was performed in technical triplicate using the ChamQ Universal SYBR qPCR Master Mix (Q711-02, Vazyme) with a Roche LightCycler96 qRT-PCR system (Roche, Germany). All data were generated using cDNA from triplicate wells for each condition. The comparative Ct method was used to calculate the relative quantity of the target gene messenger RNA (mRNA), and *GAPDH* gene was used as the internal control. The following procedures were used for qRT-PCR experiments: 30 s at 95 °C, followed by 40 cycles of 5 s at 95 °C and 30 s at 60 °C. Primer pairs are listed in Table 1.

**Table 1:** Primer sequences for qRT-PCR.

Gene	Forward primer sequence (5′→3′)	Reverse primer sequence (5′→3′)
*H-SLC7A11*	ATGCAGTGGCAGTGACCTTT	GGCAACAAAGATCGGAACTG
*M-SLC7A11*	CTGCTCGTAATACGCCCTGG	CCAGCTGACACTCGTGCTAT
*H-ALOX12*	GAGGAATTTTTGATAAGGCAGTG	CCCGACGGAGCAACTGTA
*H-ACSL4*	TCTGCTTCTGCTGCCCAATT	CGCCTTCTTGCCAGTCTTTT
*H-NRF2*	AAACCACCCTGAAACGACAG	AGCGGCTTGAATGTTTGTC
*H-p53*	ACGGTGACACGCTTCCCT	GAACGTTGTTTTCAGGAAGTAGTTT
*H-β-TRCP1*	AGTGGCCTCGGCGATTATG	GCACAGCTGTTGTATGTCTGGA
*H-GSK3β*	TGTGTGTTGGCTGAGCTGTT	CGGGGTCGGAAGACCTTAGT
*H-FFAR2*	CCGTGCAGTACAAGCTCTCC	CTGCTCAGTCGTGTTCAAGTATT
*H-GAPDH*	CAACGAATTTGGCTACAGCA	AGGGGTCTACATGGCAACTG
*M-GAPDH*	TGGCCTTCCGTGTTCCTAC	GAGTTGCTGTTGAAGTCGCA

*H* human, *M* mouse.

### Cell apoptosis and colony formation assay

Cells were treated with NaB for 24 h and then stained with YF488-Annexin V/PI Apoptosis Detection Kit (Y6002M, Suzhou, China) according to the manufacturer’s protocol. Apoptotic cells were measured by flow cytometry (BD FACSAria III, USA). For colony formation assay, the cells (1000 to 2000/well) were seeded in 6-well plates and treated with different combination of regents for 24 h, followed by culturing in RPMI-1640 or DMEM medium with 10% FBS for 7 d. Colonies were then fixed with 4% paraformaldehyde, washed with PBS and stained with crystal violet. Each assay was performed in triplicate.

### Detection of ROS and lipid ROS

HT29 cells were seeded in 6-well plates or 24-well plates and treated with NaB for 24 h. Cells were then loaded with dichlorofluorescein diacetate (DCF-DA, R252, DOJINDO, Japan) or 5 μM BODIPY 581/591 C11 (D3861, Thermo Fisher Scientific, MA, USA) at 37 °C for 30 min. After washing, cells in 6-well plates fluorescence intensity were detected by flow cytometry (BD FACSAria III, USA); cells in 24-well plates fluorescence intensity were detected by Hybrid Multi-Mode Reader (Bio-Tek, Synergy H1, USA).

### Intracellular cAMP measurement

For cAMP assay, cells were grown in 12-well plates with or without NaB treatment and treated with 100 μM IBMX for 6 h. Then cells were lysed by repeated freeze/thaw and centrifuged to remove cell debris. The supernatant was assayed for cAMP using the cAMP Parameter immunoassay kit (KGE002B, R&D Systems assay, Minneapolis, USA) according to the manufacturer’s instructions.

### Transmission electron microscopy (TEM)

HT29 cells were treated with NaB (2 mM) for 24 h and cell pellets were fixed in 2.5% electron microscopy-grade glutaraldehyde in 0.1 M sodium cacodylate buffer (pH 7.4) at 4 °C overnight. The subsequent sample preparations, including dehydration, embedding, curing, 50-nm ultrathin section preparation and staining with uranyl acetate and lead citrate were conducted as described previously. The ultrathin specimens were examined, and images were acquired by a TEM (HT7800, Japan).

### Dead cell staining assay

Cytotoxicity was determined using the dead cell staining assay test kit. To examine the effect of NaB, cells were treated with the different combination of regents for 24 h. Then the supernatant was discarded, and the cells were incubated with staining working solution (8 μM PI) for 30 min protected from light. After that, images were acquired with a fluorescence microscope (OLYMPUS CKX53, Japan).

### Tumor xenografts

Six-week-old male nude mice were obtained from Shanghai Experimental Animal Center (Shanghai, China). Randomization and blinding were used for animal studies. HT29 cells were trypsinized into single cell suspensions and resuspended in PBS. Approximately 5 × 10^6^ cells in 100 μL were injected into the nude mouse. From 7 days after injection, the diameter of the tumor was measured every 2 d by a vernier caliper. Where indicated, the NaB (30 mM) were supplemented in the drinking water and refreshed every two days throughout the experiment. Ferr-1 (10 mg/kg, every other day) [26] and MHY1485 (10 mg/kg, 2 days) [27] were administered to mice by intravenous injection and throughout the duration of experiment. Mice were sacrificed on day 28, and the tumor volume was calculated using the formula for volume: width^2^ × length × 0.5, where *L* = length, *W* = width. Tumor size must not exceed 20 mm at the largest diameter in an adult mouse, according to the IACUC. None of the experiments exceeded this limit in our study.

### Azoxymethane–DSS model of colorectal tumorigenesis

Male mice used were at the age of six weeks. Randomization and blinding were used for animal studies. Mice were injected intraperitoneally with 10 mg of AOM (sigma) per kg body weight. After 5 d, 2.5% DSS was given in the drinking water for 6 days followed by regular drinking water for 2 weeks. This cycle was repeated twice with 2% DSS. Where indicated, the NaB (30 mM) were supplemented in the drinking water and refreshed every two days. Mice were sacrificed on day 65, and the tumors were counted and photographed. Total number of tumors per colon in mice were analyzed.

### Statistical analysis

Statistical analysis was conducted with Prism 8.0 software (GraphPad). Flow cytometric data analysis was conducted using FlowJo software (BD Biosciences). All animals and samples that were successfully tested were included in our analysis, and all experiments were repeated at least triple. Data are presented as the mean ± SEM. Statistical tests included unpaired one-tailed or two-tailed Student’s *t*-test and one-way analysis of variance. A *p* value of 0.05 was considered statistically significant. In the graphed data ns, ∗, ∗∗ and ∗∗∗ respectively denote *p* values of > 0.05, < 0.05, < 0.01, and < 0.001.

## Results

### Induction of cell death by butyrate in the presence of ferroptosis inducers

**Fig. 1:**
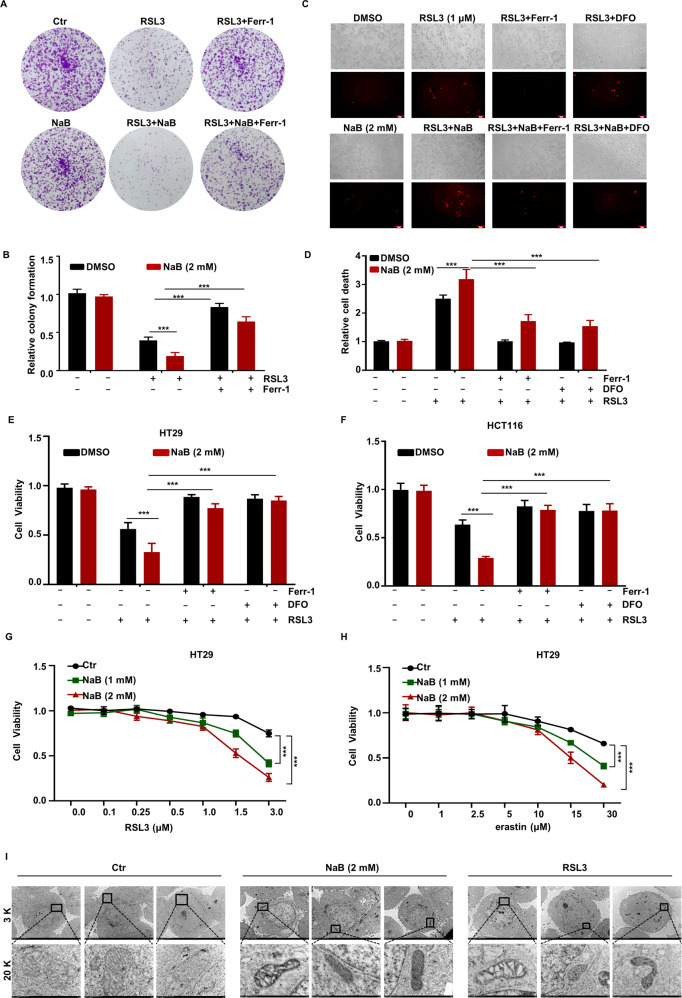
Induction of cell death by butyrate in the presence of ferroptosis inducers. HT29 cells were treated with NaB (2 mM) for 24 h in combination with RSL3 (1 μM) or Ferr-1 (5 μM), after which cell viability was detected by colony formation assay (**A**) and the quantitative data is presented (**B**). HT29 cells were treated with NaB (2 mM) for 24 h in combination with RSL3 (1 μM), Ferr-1 (5 μM), or DFO (100 μM) after which cell viability was detected by dead cell staining assay (**C**) and the quantitative data is presented (**D**). HT29 (**E**) or HCT116 (**F**) cells were treated with RSL3 (1 μM) for 6 h in combination with NaB (2 mM), Ferr-1 (5 μM) or DFO (100 μM), and the viability of indicated cells was examined using CCK-8. HT29 cells were treated with different concentrations of RSL3 for 6 h (**G**) or erastin for 20 h (**H**) in combination with indicated concentrations of NaB, and the cells viability was examined using CCK-8. **I** HT29 cells were treated with the RSL3 (1 μM) or NaB (2 mM) for 24 h, and analyze the ultrastructure of mitochondria with transmission electron microscopy.

To explore the role of SCFAs in ferroptosis, the viability of cells co-treated with SCFAs (acetate, propionate, and butyrate) and the ferroptosis inducer RSL3 was assessed. As shown in Fig. S1A, co-treatment with sodium butyrate (NaB) and RSL3 for 6 h led to a significant increase in RSL3-induced death of HT29 cells. Then, HT29 cells were treated with NaB, and decreased cell viability was observed at 1.0 and 2.0 mM (Fig. S1B). To further demonstrate the role of NaB in ferroptosis, we investigated the effect of NaB on the ability of RSL3 to induce cells ferroptosis by using a cell colony formation and dead cell staining assay. Notably, NaB significantly enhanced RSL3-induced inhibition of cell colony formation and cell survival (Figs. 1A–D and S1C–E). To further determine whether cell death promoted by NaB involves ferroptosis, we treated the cells with ferroptosis inhibitor ferrostatin-1 (Ferr-1), deferoxamine (DFO), and found that Ferr-1 not only restored cell death induced by RSL3, but also significantly blocked cell death induced by co-treatment with NaB and RSL3 in HT29 and HCT116 cells (Figs. 1A–F and S1C–E). Moreover, we investigated whether NaB regulated the sensitivity of cells to ferroptosis. The results showed that combined treatment with various concentrations of NaB and RSL3 significantly reduced cell viability in HT29 and HCT116 cells (Figs. 1G and S1F). As erastin is also known to be a ferroptosis inducer, we treated HT29 and HCT116 cells with erastin for 20 h. Erastin treatment reduced cell viability by 35%, and NaB enhanced erastin-induced cell death in a dose-dependent manner (Figs. 1H and S1G). We next determined whether NaB affected the ferroptosis in noncancer (HEK293T) cells and found that NaB treatment led to a significant increase in RSL3-induced ferroptosis (Fig. S1H).

There is accumulating evidence that the mechanisms of butyrate cytotoxicity include autophagy and apoptosis. Therefore, we examined whether apoptosis and autophagy are induced by NaB. Consistent with the findings of a previous study, NaB was found to promote autophagy, based on the protein expression of the autophagy markers p62 and LC3II in response to various concentrations of NaB and treatment times (Fig. S1I, J). As shown in Fig. S1K, treatment with NaB also induced apoptosis.

Considering that ferroptosis is characterized by mitochondrial ultrastructure changes [28], we examined the effects of NaB on cell mitochondria by performing ultrastructure analysis of mitochondria with transmission electron microscopy. In the NaB-treated cells, the mitochondria ultrastructure micrographs showed that a significant number of mitochondria exhibited disorganization of their cristae (Fig. 1I). These results suggest that butyrate plays a vital role in ferroptosis.

### Promotion of ferroptosis by butyrate via increasing lipid ROS production

**Fig. 2:**
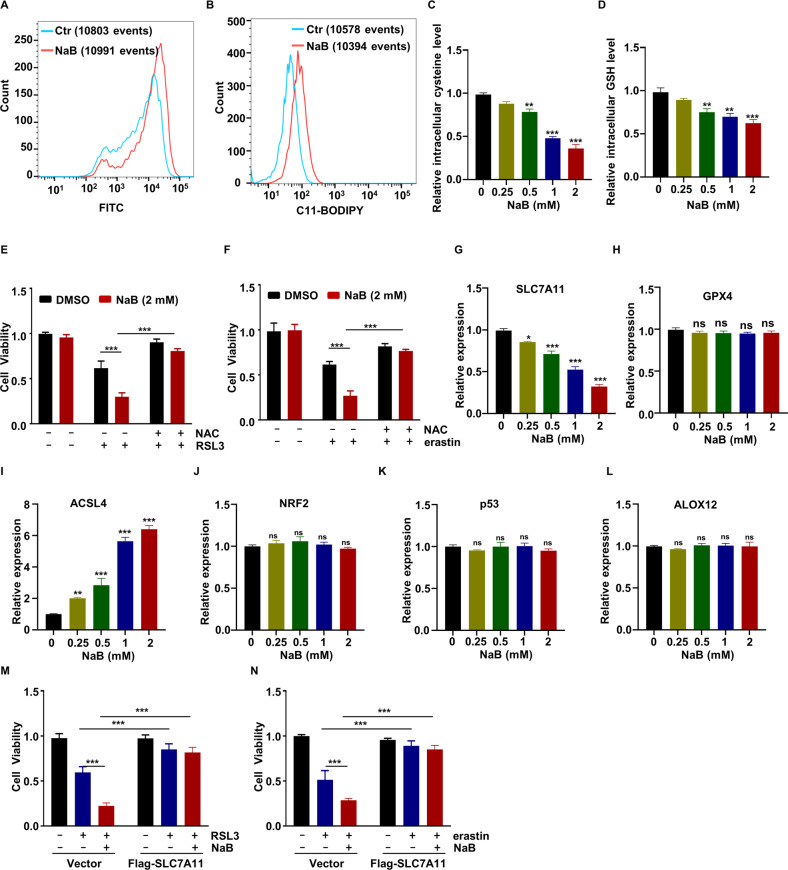
Promotion of ferroptosis by butyrate via increase in lipid ROS production and decrease in the expression of SLC7A11. HT29 cells were treated with NaB (2 mM) for 24 h, ROS production (**A**) and lipid ROS levels (**B**) were determined using flow cytometer. HT29 cells were treated with different concentrations of NaB for 6 h, and relative intracellular cysteine (**C**) and GSH (**D**) levels were showed. HT29 cells were treated with RSL3 (1 μM) (**E**) or erastin (10 μM) (**F**) in combination with NaB and NAC (2 mM) for 6 h or 20 h, and the cell viability was examined using CCK-8. HT29 cells were treated with NaB for 6 h, and the expression of SLC7A11 (**G**), GPX4 (**H**), ACSL4 (**I**), NRF2 (**J**), p53 (**K**), and ALOX12 (**L**), were measured using qRT-PCR. Vector and Flag-SLC7A11-overexpressing cells were treated with RSL3 (**M**) or erastin (**N**) in combination with NaB (2 mM) for 6 h or 20 h, followed by cell viability analysis.

It is well established that ROS plays a vital role in ferroptosis. Therefore, we next investigated whether butyrate regulated the production of ROS. The ROS level in HT29 cells treated with NaB for 24 h was measured by DCF-DA staining. As expected, NaB treatment led to a significant increase in cellular ROS levels in a dose-dependent manner (Figs. 2A and S2A), and similar results were obtained for HCT116 cells (Fig. S2B, C). As ferroptosis involves lipid peroxidation-dependent cell death, we next examined the lipid ROS levels in HT29 cells treated with NaB for 24 h by C11 BODIPY staining. As shown in Figs. 2B and S2D, NaB treatment increased lipid peroxidation.

Next, we examined the effects of butyrate on cysteine uptake and GSH levels. Consistently, NaB treatment was found to induce an decrease in intracellular cysteine and GSH levels in HT29 and HCT116 cells (Figs. 2C, D and S2E, F). To strengthen this finding, we examined the contribution of NaB-induced lipid ROS production to ferroptosis. To this end, cells were treated with NAC prior to NaB and RSL3 exposure. NAC not only inhibited RSL3-induced cell death but also significantly blocked cell death induced by RSL3/NaB co-treatment (Fig. 2E). In line with this result, NAC treatment was found to restore the phenotype and effect of erastin or combined treatment with erastin/NaB on HT29 cell viability (Fig. 2F). NaB-induced cytotoxicity is, thus, completely reversed by treatment with NAC in HT29 cells. This finding indicates that butyrate promotes ferroptosis by increasing lipid ROS production.

### Promotion of ferroptosis by butyrate via a decrease in the expression of SLC7A11

Given the critical role of SLC7A11, ACSL4, GPX4, ALOX12, NRF2, p53, and ALOX12 in ferroptosis [29], we examined whether NaB mediated ferroptosis via these genes. After treatment with NaB, cellular expression of the abovementioned genes was analyzed by qRT-PCR (Figs. 2G–L and S2G–L). Consistent with previously published results [8], the expression of ACSL4 was increased in response to NaB stimulation (Figs. 2I and S2I). In line with our results for intracellular ROS, cysteine, and GSH levels, we found that the expression of SLC7A11 significantly decreased in a time-dependent manner (Figs. 2G and S2G). Next, we examined the contribution of SLC7A11 to NaB-induced ferroptosis. Flag-SLC7A11 was overexpressed in HT29 cells, and the cells were treated with NaB or RSL3. Our results showed that Flag-SLC7A11 not only inhibited RSL3-induced cell death but also significantly blocked cell death induced by RSL3/NaB co-treatment (Fig. 2M). In line with this result, Flag-SLC7A11 overexpression restored the effect of erastin or combined treatment with erastin/NaB on HT29 cells viability (Fig. 2N). These findings indicate that the expression of SLC7A11 plays an important role in butyrate-induced ferroptosis.

### Butyrate-induced SLC7A11 downregulation via the GSK3β-β-TRCP1-NRF2 pathway

**Fig. 3:**
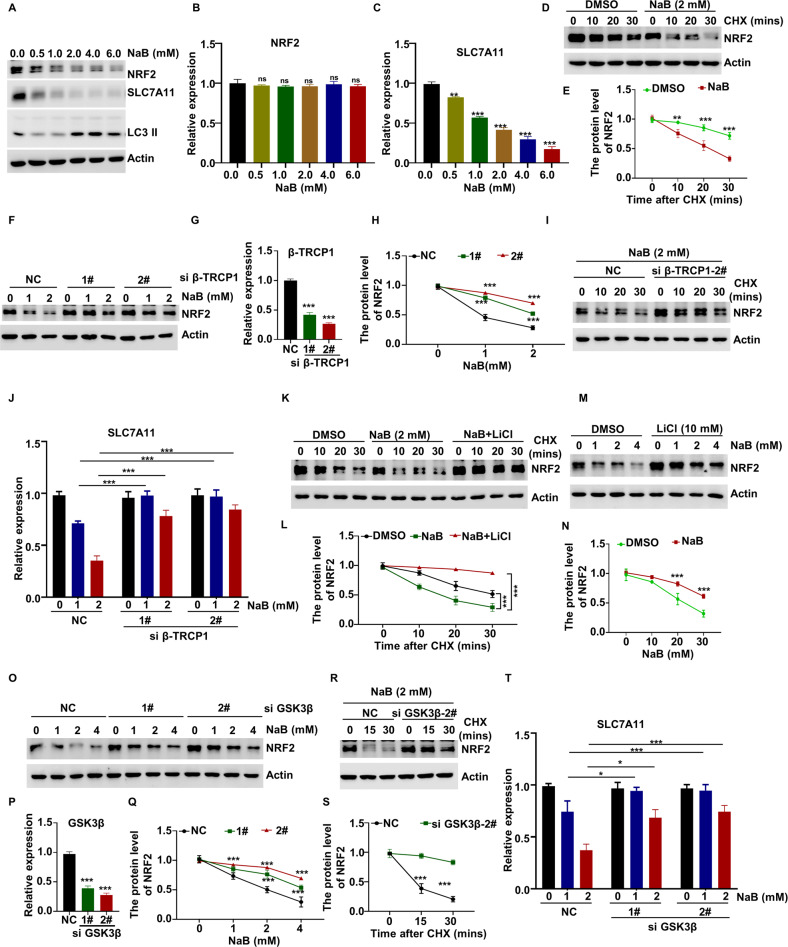
Butyrate-induced SLC7A11 downregulation via the GSK3β-β-TRCP1-NRF2 pathway. HT29 cells were treated with the indicated concentrations of NaB or in combination with bafilomycin A1 for 6 h, the levels of the indicated proteins were evaluated by WB (**A**), the expression of NRF2 (**B**) and SLC7A11 (**C**) were measured using qRT-PCR. HT29 cells were treated with CHX for the indicated time after pretreatment with NaB for 6 h, and then WB was used to evaluate the levels of NRF2 (**D**). Quantitative data for NRF2 protein levels are presented (**E**). **F**–**H** β-TRCP1-knockdown HT29 cells were treated with the indicated concentrations of NaB for 6 h, and then WB was used to evaluate the levels of NRF2 (**F**) and quantitative data for NRF2 protein levels are presented (**H**). The knockdown efficiency was measured by qRT-PCR (**G**). **I** β-TRCP1-knockdown HT29 cells were treated with CHX for the indicated time after pretreatment with NaB for 6 h, and then WB was used to evaluate the levels of NRF2. The knockdown efficiency was shown in (**G**). **J** β-TRCP1-knockdown HT29 cells were treated with the indicated concentrations of NaB for 6 h, and the expression of SLC7A11 was measured using qRT-PCR. The knockdown efficiency was shown in (**G**). **K**, **L** HT29 cells were treated with CHX for the indicated time after pretreatment with NaB or in combination with LiCl (10 mM) for 6 h, and then WB was used to evaluate the levels of NRF2 (**K**). Quantitative data for NRF2 protein levels are presented (**L**). **M**, **N** HT29 cells were treated with the indicated concentrations of NaB or in combination with LiCl, and then WB was used to evaluate the levels of NRF2 (**M**). Quantitative data for NRF2 protein levels are presented (**N**). **O**–**Q** GSK3β-knockdown HT29 cells were treated with the indicated concentrations of NaB for 6 h, and then WB was used to evaluate the levels of NRF2 (**O**). Quantitative data for NRF2 protein levels are presented (**Q**) and the knockdown efficiency was measured by qRT-PCR (**P**). GSK3β-knockdown HT29 cells were treated with CHX for the indicated time after pretreatment with NaB for 6 h, and then WB was used to evaluate the levels of NRF2 (**R**), quantitative data for NRF2 protein levels are presented (**S**). The knockdown efficiency was shown in (**P**). **T** GSK3β-knockdown HT29 cells were treated with the indicated concentrations of NaB for 6 h, and the expression of SLC7A11 was measured using qRT-PCR. The knockdown efficiency was shown in (**P**).

We sought to further elucidate the molecular mechanisms by which SLC7A11 expression is inhibited by butyrate. The involvement of SLC7A11 in NaB-induced ferroptosis was examined by WB analysis. In line with the qRT-PCR results, NaB treatment decreased SLC7A11 protein expression in a dose-dependent manner (Figs. 3A, C and S3A, C, D, F, G, I); this indicates that NaB exposure inhibits the expression of SLC7A11. As NRF2 acts upstream of SLC7A11 [30], we also measured the protein level of NRF2 in NaB-treated cells. We found that NaB treatment led to a significant decrease in NRF2 protein levels (Figs. 3A and S3A, D, G). In contrast, the levels of NRF2 transcripts remained relatively stable in response to NaB treatment (Figs. 3B and S3B, E, H); this indicates that butyrate regulates the protein level of NRF2 at the posttranscriptional level. To further demonstrate that butyrate promotes the degradation of NRF2, we co-treated HT29 cells with NaB and cycloheximide (CHX), which blocks new protein synthesis, and determined the effect on NRF2 stability. As shown in Fig. 3D, E, compared with the control cells, pretreatment with NaB significantly decreased CHX-mediated NRF2 instability; this indicates that butyrate promotes NRF2 degradation through the ubiquitin-proteasome degradation pathway.

The stability of NRF2 has been reported to be regulated by β-TRCP1 [31]. Consistent with this finding, our data showed that β-TRCP1 deficiency dramatically delayed the NaB-induced degradation of NRF2 (Fig. 3F–I). In line with this, we also found that NaB-inhibited SLC7A11 expression is rescued in β-TRCP1-knockdown cells (Figs. 3J and S3J, K). Previous studies have shown that NRF2 is degraded through β-TRCP1-mediated ubiquitination in a glycogen synthase kinase-3 (GSK3β)-dependent manner [20, 31]. Thus, we next examined whether GSK3β has any effect on NRF2 degradation. Our data showed that LiCl, an inhibitor of GSK3β, markedly increased the protein levels of NRF2 in response to NaB treatment (Fig. 3K–N). In addition, the stability of NRF2 induced by NaB was significantly abolished in GSK3β-knockdown cells (Fig. 3O–S). We also examined whether GSK3β affects the expression of SLC7A11, and our data showed that GSK3β knockdown markedly restored NaB-induced downregulation of SLC7A11 (Figs. 3T and S3L, M). These findings indicate the GSK3β-β-TRCP1-induced NRF2 degradation is important for NaB-dependent SLC7A11 expression. Collectively, these results demonstrate that the inhibition of SLC7A11 expression by butyrate was dependent on the GSK3β-β-TRCP1-NRF2 pathway.

### Promotion of ferroptosis by butyrate via the mTORC2-AKT-GSK3β pathway

**Fig. 4:**
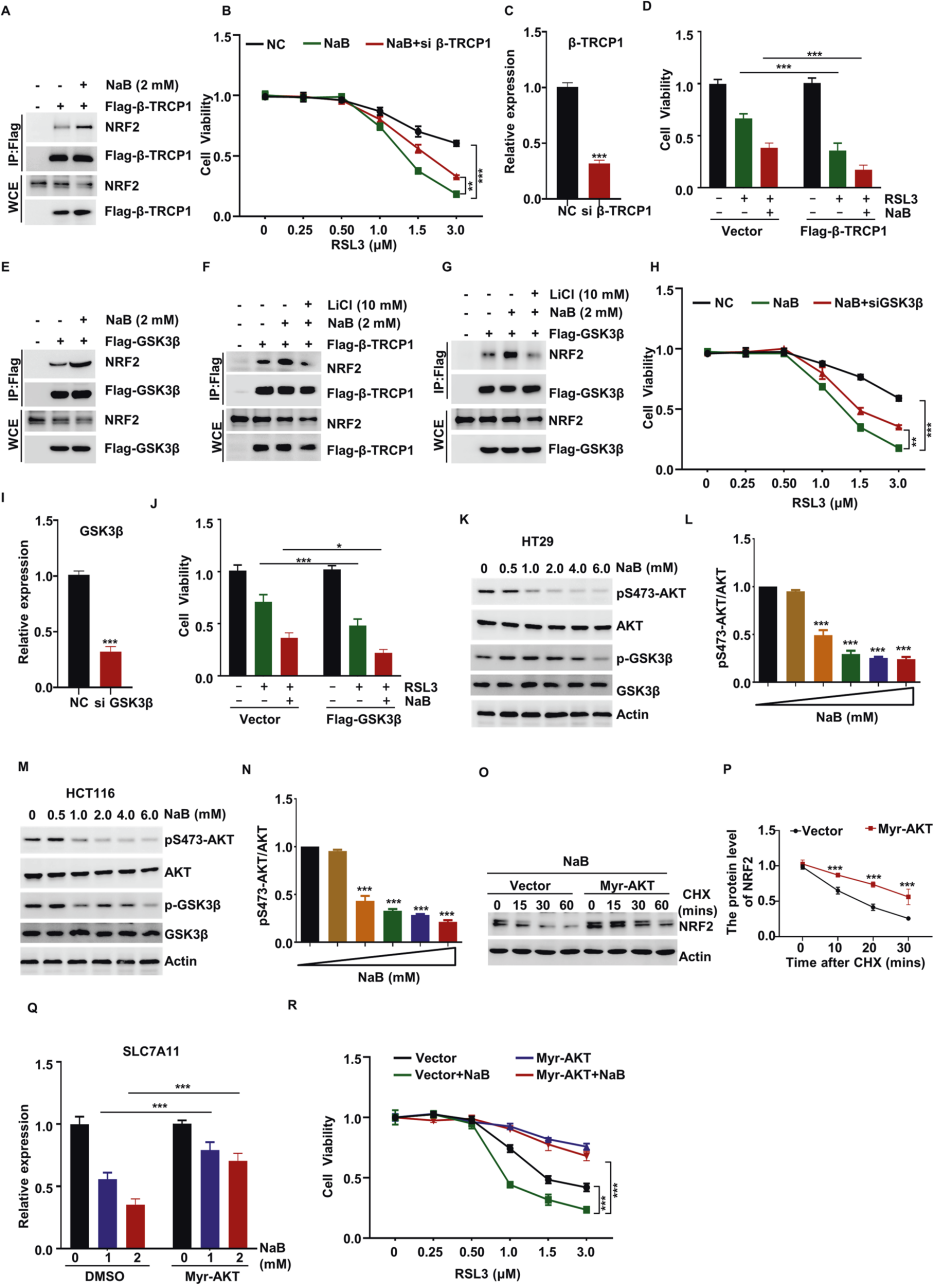
Promotion of ferroptosis by butyrate via the mTORC2-AKT-GSK3β pathway. **A** Flag-β-TRCP1 was overexpressed in HEK293T cells, and cells were treated with NaB for 6 h, the interaction between Flag-β-TRCP1 and NRF2 was detected by Co-IP assay, and the indicated protein were detected by WB. β-TRCP1-knockdown HT29 cells were treated with different concentrations of RSL3 for 6 h in combination with NaB, and the cell viability was examined using CCK-8 (**B**), the knockdown efficiency was measured by qRT-PCR (**C**). **D** Flag-β-TRCP1 overexpressed HT29 cells were treated with RSL3 or in combination with NaB for 6 h, the cell viability was examined using CCK-8. **E** Flag-GSK3β was overexpressed in HEK293T cells, and cells were treated with NaB for 6 h, the interaction between Flag-GSK3β and NRF2 was detected by Co-IP assay, the indicated proteins were detected by WB. Flag-β-TRCP1 (**F**) or Flag-GSK3β (**G**) was overexpressed in HEK293T cells, and cells were treated with NaB or in combination with LiCl for 6 h, the interaction between Flag-β-TRCP1 (**F**) or Flag-GSK3β (**G**) and NRF2 was detected by Co-IP assay, and the indicated proteins were detected by WB. GSK3β-knockdown HT29 cells were treated with different concentrations of RSL3 for 6 h in combination with NaB, and the cell viability was examined using CCK-8 (**H**), the knockdown efficiency was measured by qRT-PCR (**I**). **J** Flag-GSK3β overexpressed HT29 cells were treated with RSL3 or in combination with NaB for 6 h, and the cells viability was examined using CCK-8. HT29 (**K**, **L**) or HCT116 (**M**, **N**) cells were treated with the indicated concentrations of NaB for 6 h, the levels of pS473-AKT, p-GSK3β and the indicated proteins were evaluated by WB (**K**, **M**). Quantitative data for pS473-AKT/AKT are presented (**L**, **N**). Myr-AKT overexpressed HT29 cells were treated with CHX for the indicated time after pretreatment with NaB for 6 h, and then WB was used to evaluate the levels of NRF2 (**O**), quantitative data for NRF2 protein levels are presented (**P**). **Q** Myr-AKT overexpressed HT29 cells were treated with the indicated concentrations of NaB for 6 h, the expression of SLC7A11 was measured using qRT-PCR. **R** Myr-AKT overexpressed HT29 cells were treated with different concentrations of RSL3 for 6 h in combination with NaB, and the cells viability was examined using CCK-8.

To understand the mechanism by which butyrate regulates NRF2 degradation, we examined whether butyrate affected the interaction between NRF2 and β-TRCP1 through a Co-IP assay. We found that the binding of NRF2 to β-TRCP1 was markedly increased in response to NaB stimulation (Fig. 4A). Next, we examined the effect of β-TRCP1 on sensitivity of cells to ferroptosis, and we found that NaB- and RSL3-induced ferroptosis was gradually restored in β-TRCP1-deficient cells (Fig. 4B, C), while β-TRCP1 overexpression partly sensitized HT29 cells to ferroptosis induced by NaB and RSL3 (Fig. 4D). Accordingly, β-TRCP1 deficiency increased the resistance of HT29 cells to NaB and erastin-induced ferroptosis (Fig. S4A, B).

With regard to the role of GSK3β in butyrate-induced NRF2 degradation, we examined whether butyrate regulated the interaction between GSK3β and NRF2 and found that the binding between GSK3β and NRF2 was significantly increased in response to NaB stimulation (Fig. 4E). We also found that LiCl exerted a marked inhibitory effect on the NaB-induced binding of β-TRCP1/GSK3β with NRF2 (Fig. 4F, G). This implies that the NaB-mediated interaction between β-TRCP1 and NRF2 is regulated by GSK3β. Consistent with this finding, we found that GSK3β knockdown protects HT29 cells from NaB- and RSL3/erastin-induced ferroptosis (Figs. 4H, I and S4C, D), while GSK3β-overexpressing HT29 cells exhibit moderate sensitivity to NaB- and RSL3-induced ferroptosis (Fig. 4J). Based on these findings, we can conclude that the GSK3β-β-TRCP1-NRF2 axis is important for butyrate-induced cell ferroptosis via upregulating the expression of SLC7A11.

GSK3β has been reported to be a substrate of AKT, which phosphorylates GSK3β and inhibits its activity [32]. Thus, we treated HT29 and HCT116 cells with different concentrations of NaB and examined the activation status of AKT-GSK3β by assessing the levels of AKT and GSK3β phosphorylation. Our results showed that NaB treatment reduces pS473-AKT and pSer9-GSK3β levels in a dose-dependent manner (Fig. 4K–N). To confirm this finding, we examined the effect of Myr-AKT, a constitutively active form of AKT, which considered to be modified with myristoylation on NH_2_-terminally [33], and constitutively stimulates downstream signaling. We found that Myr-AKT overexpression significantly enhanced pS473-AKT level even in the case of NaB treatment (Fig. S4E) and well delayed the NaB- and CHX-induced degradation of NRF2 (Fig. 4O, P). Consistent with this finding, our data showed that NaB-induced inhibition of SLC7A11 expression is rescued in Myr-AKT-overexpression cells (Fig. 4Q). In addition, we found that Myr-AKT overexpression rendered HT29 cells resistant to ferroptosis induced by NaB and RSL3 (Fig. 4R). Based on these findings, we can conclude that butyrate probably blocked the phosphorylation of GSK3β via inhibition of AKT activation, and this enhanced the activation of GSK3β and promoted the degradation of NRF2.

### Promotion of ferroptosis by butyrate via cAMP-PKA-mediated AKT-GSK3 activation

**Fig. 5:**
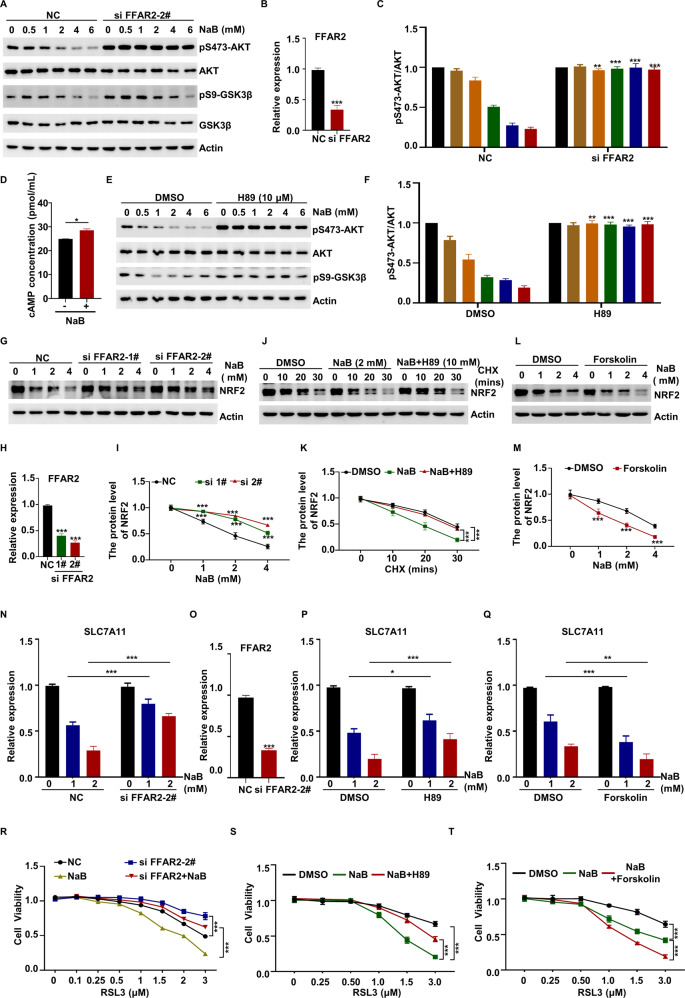
Promotion of ferroptosis by butyrate via cAMP-PKA-mediated AKT-GSK3 activation. **A**–**C** FFAR2-knockdown HT29 cells were treated with the indicated concentrations of NaB for 6 h, the levels of pS473-AKT, p-GSK3β and the indicated proteins were evaluated by WB (**A**). The knockdown efficiency was measured by qRT-PCR (**B**) and quantitative data for pS473-AKT/AKT is presented (**C**). **D** HCT116 cells were treated with the NaB for 6 h, and the cAMP level was detected. HT29 cells were treated with the indicated concentrations of NaB or in combination with H89 for 6 h, the levels of pS473-AKT, p-GSK3β and indicated the proteins were evaluated by WB (**E**) and quantitative data for pS473-AKT/AKT is presented (**F**). **G**–**I** FFAR2-knockdown HT29 cells were treated with the indicated concentrations of NaB for 6 h, and then WB was used to evaluate the levels of NRF2 (**G**). The knockdown efficiency was measured by qRT-PCR (**H**) and quantitative data for NRF2 protein levels are presented (**I**). After pretreatment treated with NaB or in combination with H89 for 6 h, HT29 cells were treated with CHX for the indicated time, and then WB was used to evaluate the levels of NRF2 (**J**), quantitative data for NRF2 protein levels were presented (**K**). HT29 cells were treated with the indicated concentrations of NaB or in combination with Forskolin for 6 h, and then WB was used to evaluate the levels of NRF2 (**L**), quantitative data for NRF2 protein levels were presented (**M**). FFAR2-knockdown HT29 cells were treated with the indicated concentrations of NaB for 6 h, and the expression of SLC7A11 was measured using qRT-PCR (**N**), the knockdown efficiency was measured by qRT-PCR (**O**). HT29 cells were treated with the indicated concentrations of NaB and in combination with H89 (**P**) or Forskolin (**Q**) for 6 h, and the expression of SLC7A11 was measured using qRT-PCR. **R** FFAR2-knockdown HT29 cells were treated with different concentrations of RSL3 or in combination with NaB, and the cell viability was examined using CCK-8. The knockdown efficiency was shown in (**O**). HT29 cells were treated with different concentrations of RSL3, NaB and in combination with H89 (**S**) or Forskolin (**T**) for 6 h, the cell viability was examined using CCK-8.

To further explore the mechanisms underlying the suppressive effect of butyrate on AKT activation and ferroptosis, we next examined whether NaB inhibited AKT-GSK3 through FFAR2, which has been reported to be a receptor of butyrate [34]. We knocked down FFAR2 using specific siRNAs, and found that knockdown of FFAR2 rescued the NaB-mediated inhibition of pS473-AKT and pSer9-GSK3β (Fig. 5A–C). Moreover, our data showed that NaB significantly enhanced cAMP levels (Fig. 5D).

Next, we considered the possibility that cAMP regulates mTOR activation. To examine this, we treated HT29 cells with different doses of forskolin (an activator of adenylate cyclase) and found that it was strongly correlated with the downregulation of pS473-AKT and pSer9-GSK3β in a dose-dependent manner (Fig. S5A). Moreover, we treated HT29 cells with the PKA inhibitor H89. Surprisingly, our data showed that H89 could markedly restore the inhibitory effect of NaB on the phosphorylation levels of pS473-AKT and pSer9-GSK3β (Figs. 5E, F and S5B). This result indicates that butyrate inhibits the AKT-GSK3 pathway in a cAMP-PKA-dependent manner.

With regard to the role of AKT-GSK3 in butyrate-induced ferroptosis, we examined whether FFAR2, H89, and forskolin regulated SLC7A11 expression and ferroptosis. We treated HT29 cells with different concentrations of NaB, and our results demonstrated that FFAR2 knockdown or H89 treatment delayed NaB- or CHX-induced degradation of NRF2 (Figs. 5G–K and S5C, D), rescued the inhibitory effect of NaB on SLC7A11 expression (Fig. 5N–P), and induced ferroptosis resistance in HT29 cells treated with NaB and RSL3 (Fig. 5R, S). Conversely, forskolin treatment had an opposite effect, as it promoted the NaB-induced degradation of NRF2 (Fig. 5L, M), further promoted NaB-mediated downregulation of SLC7A11 expression (Fig. 5Q), and increased the sensitivity of cells to NaB-induced ferroptosis (Fig. 5T). Collectively, these results indicate that cAMP-PKA-mediated AKT-GSK3 activation regulates SLC7A11 expression and cell ferroptosis.

### Promotion of ferroptosis by butyrate via inhibition of the AKT and mTORC1 pathway in a cAMP-PKA-dependent manner

**Fig. 6:**
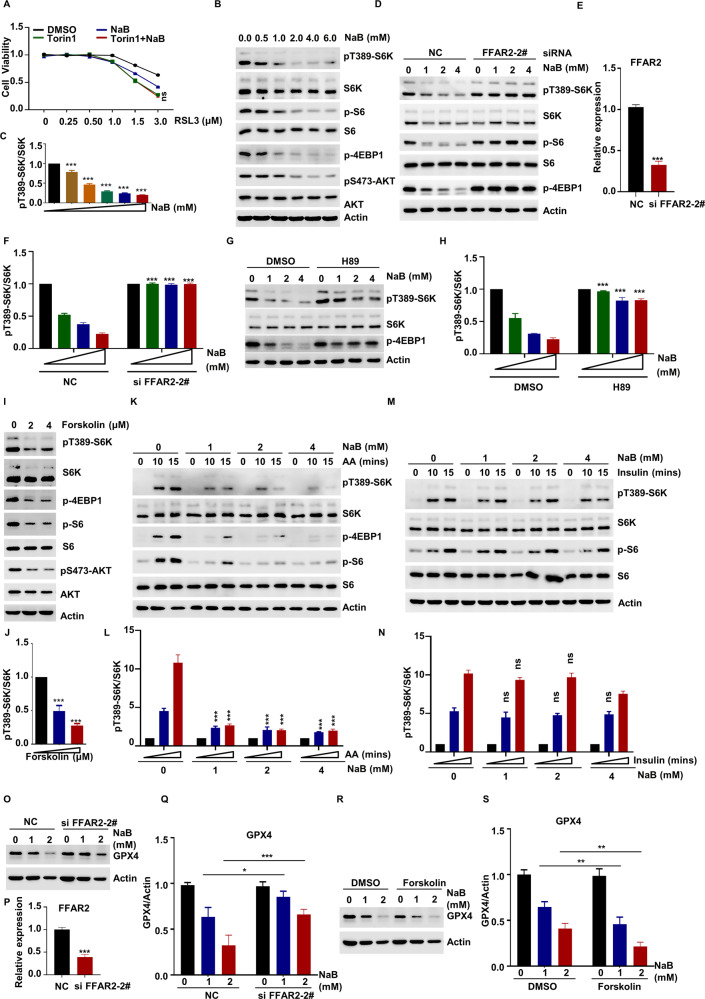
Promotion of ferroptosis by butyrate via inhibition of the AKT and mTORC1 pathway in a cAMP-PKA-dependent manner. **A** HT29 cells were treated with different concentrations of RSL3 or in combination with NaB and Torin1 for 6 h, the cell viability was examined using CCK-8. HT29 cells were treated with the indicated concentrations of NaB for 6 h, then WB was used to evaluate the levels of pT389-S6K and indicated proteins (**B**), and quantitative data for pT389-S6K/S6K is presented (**C**). **D**–**F** FFAR2-knockdown HT29 cells were treated with the indicated concentrations of NaB for 6 h, and then WB was used to evaluate the levels of pT389-S6K and indicated proteins (**D**). The knockdown efficiency was measured by qRT-PCR (**E**) and quantitative data for pT389-S6K/S6K is presented (**F**). HT29 cells were treated with NaB or in combination with H89 for 6 h, then WB was used to evaluate the levels of pT389-S6K and indicated proteins (**G**), and quantitative data for pT389-S6K/S6K is presented (**H**). HT29 cells were treated with the indicated concentrations of Forskolin for 6 h, then WB was used to evaluate the levels of pT389-S6K and indicated proteins (**I**), and quantitative data for pT389-S6K/S6K is presented (**J**). HT29 cells were stimulated with amino acid (**K**, **L**) or insulin (**M**, **N**) for the indicated time after pretreatment with indicated concentrations of NaB for 6 h, then WB was used to evaluate the levels of pT389-S6K and indicated proteins (**K**, **M**), and quantitative data for pT389-S6K/S6K are presented (**L**, **N**). **O**–**Q** FFAR2-knockdown HT29 cells were treated with the indicated concentrations of NaB for 6 h, and the protein level of GPX4 was measured using WB (**O**). The knockdown efficiency was measured by qRT-PCR (**P**) and quantitative data for GPX4 protein levels were presented (**Q**). HT29 cells were treated with indicated concentrations of NaB or in combination with Forskolin for 6 h, and the protein level of GPX4 was measured using WB (**R**), quantitative data for GPX4 protein levels were presented (**S**).

A recent study showed that mTORC1 inhibited ferroptosis by promoting GPX4 protein synthesis [35]. Next, we examined the role of mTORC1 in regulating NaB-dependent ferroptosis. We treated the cells with the mTOR inhibitor Torin1, which blocks the activation of both mTORC1 and mTORC2, and found that NaB treatment did not further aggravate the inhibitory effects of Torin1 on cell viability (Fig. 6A), indicating NaB-induced mTOR-dependent ferroptosis. Further results showed that NaB reduces pT389-S6K, p-4EBP1, and p-S6 levels in a dose-dependent manner (Figs. 6B, C and S5E, F). Consistent with this finding, FFAR2 knockdown blocked the ability of NaB to inhibit the phosphorylation of S6K, 4EBP1, and S6 in HT29 cells (Fig. 6D–F). Similar results were obtained for H89-treated cells (Fig. 6G, H). In contrast, the activation of mTORC1 was significantly downregulated by forskolin treatment (Fig. 6I, J). This finding indicates that cAMP-PKA plays an important role in NaB-induced mTORC1 inactivation.

Because mTORC1 activation is closely associated with environmental stimuli such as amino acid- and insulin-related signals [36], we explored whether butyrate treatment could affect mTORC1 activation mediated by these two factors. Our data showed that NaB treatment could block the amino acid-induced increase in the phosphorylation levels of S6K, 4EBP1, and S6 (Fig. 6K, L), but did not affect insulin-mediated activity (Fig. 6M, N).

We next examined whether NaB-inhibited mTORC1 affects the synthesis of GPX4. Our results showed that GPX4 protein synthesis was significantly inhibited in response to NaB stimulation and the inhibitory effect can be abolished by FFAR2 knockdown (Fig. 6O–Q). In contrast, forskolin treatment enhanced the inhibitory effect of NaB on the protein level of GPX4 (Fig. 6R, S). This suggests that the NaB-mediated suppression of GPX4 synthesis occurs in a FFAR2-cAMP-PKA-dependent manner. Collectively, these results demonstrate that the recognition of NaB by FFAR2 leads to inhibition of the AKT and mTORC1 pathway in a cAMP-PKA-dependent manner, and this promotes ferroptosis via regulation of the level of SLC7A11 and GPX4.

### Butyrate regulates the development of colorectal cancer in an mTOR- and ferroptosis-dependent manner

**Fig. 7:**
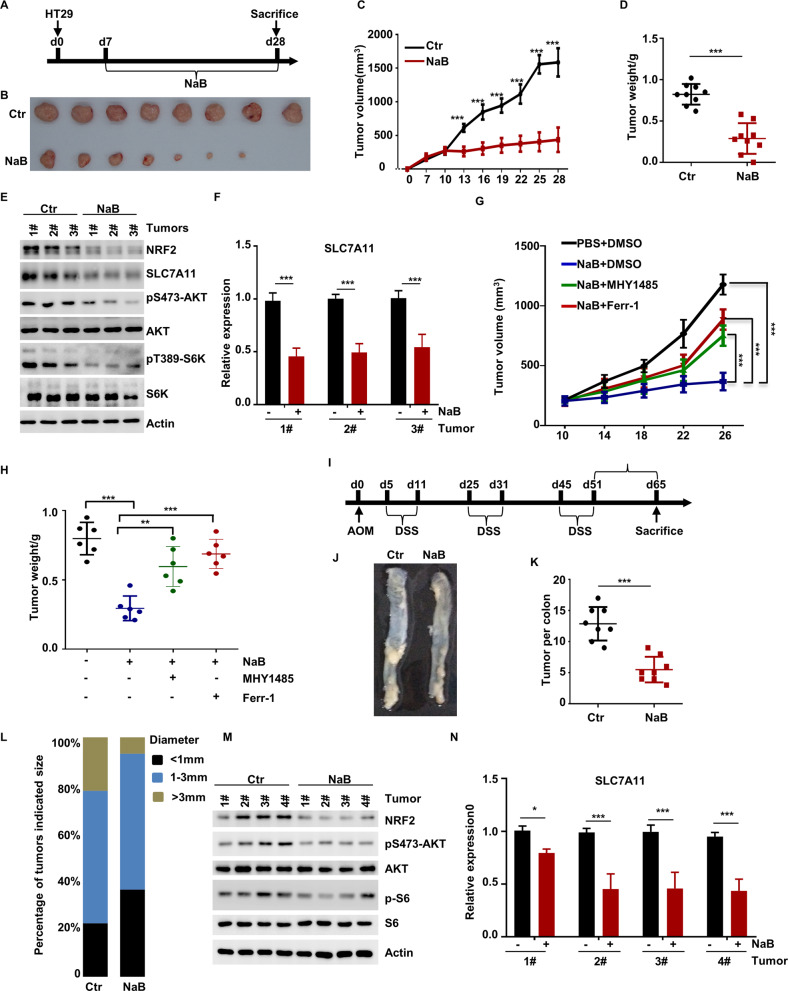
Butyrate regulates the development of colorectal cancer in an mTOR- and ferroptosis-dependent manner. **A**–**D** HT29 tumor cells growth via xenograft model (*n* = 8 per group). The diameter of the tumor was measured every 2 days after 7 days of injection (**A**). Tumors were obtained on the 28th day after injection (**B**), and the volume (**C**) and weight (**D**) of the tumors were measured. **E** mTOR activation, the protein level of NRF2 and SLC7A11 was tested using WB in the tumor samples. **F** The expression of SLC7A11 was tested using qRT-PCR in the tumor samples. **G** HT29 cells were injected into nude mice subcutaneous, NaB (30 mM) were supplemented in the drinking water and refreshed every two days throughout the experiment. Ferr-1 and MHY1485 were administered by intravenous injection and throughout the duration of experiment (*n* = 6 per group). The diameter of the tumor was measured after 7 days of injection. Tumors were obtained on the 28th day after injection. The tumors volume (**G**) and tumors weight (**H**) were measured. **I** Experimental design. **J** Representative images of colon tumors in mice on the 65th day after injection of azoxymethane. Number (**K**) and size (**L**) of colon tumors in Ctr (*n* = 8) and NaB treatment (*n* = 8) mice. **M** mTOR activation, the protein level of NRF2 was tested using WB in the tumor samples. **N** The expression of SLC7A11 was tested using qRT-PCR in the tumor samples.

We next examined whether NaB is involved in mTOR- and ferroptosis-mediated colorectal cancer tumorigenesis in vivo. Our data showed that the HT29 cells treated with NaB exhibited a weak ability to form tumor with a slow growth rate (Fig. 7A–D). WB for pS473-AKT and pT389-S6K demonstrated that NaB treatment resulted in the mTOR inactivation in tumors (Fig. 7E). In an attempt to clarify the biological significance of NaB in ferroptosis, we analyzed the expression levels of SLC7A11 in tumors and found that NaB treatment downregulated the expression levels of SLC7A11 (Fig. 7F), as well as the stability of NRF2 (Fig. 7E). More importantly, we found that the inhibitory effect of NaB on tumor growth could be eliminated by administrating MHY1485 (mTORC1 activator) and Ferr-1 (ferroptosis inhibitor) to mice throughout the duration of the experiment (Fig. 7G, H). Altogether, these findings suggest that NaB regulates tumor activity by inhibiting the activation of the mTOR pathway and promoting ferroptosis.

To confirm that NaB is involved in the development of colorectal cancer, we investigated the role of NaB in colorectal cancer using the established mouse model of colitis-associated colorectal tumorigenesis. We intraperitoneally injected mice with azoxymethane followed by three rounds of DSS treatment (Fig. 7I). By counting the number of tumors in mouse colons, we found that NaB-treated mice developed fewer tumor colonies compared to control group mice (Fig. 7J–L). Consistently, mTORC1 activity was decreased in the tumor samples from NaB-treated mice (Fig. 7M). In addition, the stability of NRF2 was reduced in the NaB-treated tumors (Fig. 7M), as well as the expression of SLC7A11 (Fig. 7N). In summary, these data indicate that NaB plays important role in development of colorectal cancer via regulating the mTOR activation.

## Discussion

In the current study, we set out to explore the role of butyrate in regulating ferroptosis and tumor growth. We found that NaB, via the FFAR2-cAMP-PKA pathway, promoted ferroptosis by increasing the cellular ROS levels and downregulating the GSH levels, and that the AKT-GSK3-NRF2 and mTORC1 pathway played an essential role in butyrate-mediated SLC7A11 and GPX4 expression. More importantly, the NaB treatment is correlated to the mTOR-dependent ferroptosis and consequent tumor growth, implicating the potential clinical applications of NaB for future cancer treatments. In our results, we found that the far more pronounced effects in vivo than in vitro on ferroptosis of NaB. The most reasonable explanation about this might be the difference in duration of treatment and NaB concentration. Through these findings, we have uncovered a previously unappreciated signaling pathway that is critical role of butyrate in ferroptosis and colorectal cancer.

Interestingly, the effect of butyrate on cellular metabolism varies widely by cell type. For example, butyrate can stimulate the proliferation of normal colon cells, but not colon cancer cells. This differential effect is also known as the “butyrate paradox” [37], and could be explained by the role of butyrate as an HDAC inhibitor in cancer cells and as an activator of histone acetyltransferase 1 (HAT1) in normal cells [38]. Moreover, the anti-inflammatory effects of butyrate have also been implicated in the NF-kB pathway and monocyte- and neutrophil-mediated anti-inflammatory cytokine production [39–41]. Furthermore, butyrate can promote the memory potential of CD8+ T cells [42], and is also known to inhibit the maturation of dendritic cells and, thereby, regulate the differentiation of dendritic cells derived from human monocytes [43]. In the current study, we discovered a new regulatory function involving ferroptosis that underlies butyrate-induced cell viability. Our finding demonstrates the negative regulatory effect of butyrate in development of colorectal cancer. However, our findings differ from those of previous studies, as we found that butyrate promotes ferroptosis, not anti-inflammatory, by inhibiting the activities of AKT and mTORC1, rather than inhibiting the activity of HAT1.

Accumulating evidence suggests that apoptosis and autophagy are the mechanisms by which butyrate inhibits cell viability. Consistent with these results, we found that butyrate also inhibits autophagy and apoptosis (Fig. S1H–J). Moreover, in this study, we demonstrated that NaB can promote ferroptosis in HT29 cells through its effects on ferroptosis inducers, ferroptosis inhibitors, and sensitivity to ferroptosis. Zhao et al. reported that butyrate treatment enhanced expression of ACSL4, which makes cells more susceptible to lipid peroxidation and leads to ferroptosis [8]. In our study, we not only confirmed that butyrate promoted the expression of ACSL4, but also revealed a new mechanism of butyrate in ferroptosis involving inhibition of the expression of SLC7A11 and GPX4—which is also an important mechanism for butyrate-mediated ferroptosis. Some studies indicated that SLC7A11 overexpression inhibited RSL3-induced lipid peroxidation and ferroptosis [35, 44–46]. The likely explanation for this involves: (1) SLC7A11 overexpression increases cystines level and increased intracellular cystines can protect against ferroptosis independently of GPX4 by supplying sulfur to S^0^ biosynthetic pathways (formed Hydropersulfides) [46]. And Hydropersulfides (RSSH) can be employed to inhibit lipid peroxidation via their action as radical-trapping antioxidants and inhibit ferroptosis induced by either GPX4 inhibition or GPX4 deletion [45]. (2) SLC7A11 could regulates GPX4 protein levels and modulates ferroptosis sensitivity to RSL3 partly through regulating GPX4 protein levels. The results showed that SLC7A11-overexpressing cells at least partially reduced sensitivity of cells to RSL3 [35]. Therefore, preserved expression of SLC7A11 could protect against RSL3.

The mitochondrion play an important role in ferroptosis [28]. Firstly, the mitochondrion is a major source of cellular ROS [47]. Specifically, the electron leakage from ETC complexes I and III produces hydrogen peroxide (H_2_O_2_). Then H_2_O_2_ can react with Fe^2+^ to generate hydroxyl radicals (•OH), which then promote form PUFAs to PUFA hydroperoxides (PUFA-OOH) [47, 48]. In addition, the mitochondrion is also the major organelle to generate ATP. Recent studies showed that cells lacking of ATP activates the energy sensor AMP-activated protein kinase (AMPK), which potently suppresses the synthesis of some PUFAs and ferroptosis through phosphorylating and inactivating acetyl-CoA carboxylase [49, 50]. More importantly, various metabolic activities render mitochondria particularly susceptible to lipid peroxidation; therefore, cells have built up strong defense systems against ferroptosis in mitochondria [51]. For example, upon GPX4 inactivation, dihydroorotate dehydrogenase (DHODH, a mitochondrial enzyme) steps in to quench lipid peroxides and defend against ferroptosis in mitochondria [52]. Overexpression of mitochondrial ferritin, an iron-storage protein localized in mitochondria, was shown to suppress erastin-induced ferroptosis likely via promoting iron storage in mitochondria [53]. There is study that show mitochondria plays a crucial and proactive role in cysteine deprivation-induced ferroptosis [54]. In our study, NaB not only caused a decrease of intracellular cysteine level, but also induced lipid ROS production via suppressing the expression of SLC7A11 and GPX4. Notably, NaB treatment disorganized the cristae of mitochondria. Therefore, we suggested that mitochondria act a crucial role in NaB-induced ferroptosis.

NRF2 is a key regulatory factor required for cells to maintain a steady oxidative state [55], and its plays a role in ferroptosis regulation [56–58]. In accordance with previous reports, our data demonstrated that NRF2 stability involved NaB-mediated ferroptosis. In addition, we found that NaB promotes the elevation of intracellular cAMP levels through FFAR2, and that NaB inhibits the phosphorylation of AKT-GSK3 in a cAMP-PKA-dependent manner. Further, phosphorylation of GSK3 inhibits its activity and promotes the binding of βTRCP1 to NRF2, ultimately triggering the degradation of NRF2. This is also an important mechanism underlying butyrate-mediated decrease in SLC7A11 expression and GSH synthesis, and increase in ROS and ferroptosis.

Butyrate acts as an extracellular stimulus in intracellular activities, and this raises the question of how an extracellular component mediates intracellular signal transduction. Interestingly, FFAR2 receptors are the primary receptors for butyrate [34]. In our study, we found that deletion of FFAR2 not only inhibited the inhibitory effect of NaB on AKT, GSK3, and mTOR, but also restored the inhibitory effect of NaB on the expression of SLC7A11 and GPX4, as well as ferroptosis. Moreover, we demonstrated that the effect of NaB was mainly achieved via the cAMP-PKA pathway. However, the details of the molecular mechanism need to be further researched.

A recent study showed that rapamycin and Torin1 treatment can decrease GPX4 protein levels [24, 35]. Consistent with these findings, in our study, we found that NaB, via inhibition of mTORC1 activation, obviously affected the GPX4 protein levels. The phosphorylation of 4EBP1 plays an important role in suppressing GPX4 protein synthesis [35], and our results showed that NaB blocked the amino acid-induced increase in the phosphorylation levels of 4EBP1. However, another study has shown that mTORC1 negatively regulates the expression of GPX4 protein [59]. Thus, the specific context and mechanisms by which mTORC1 regulates GPX4 protein synthesis remain unclear, and further studies are needed to clarify these regulatory mechanisms.

As mTORC1 activation is closely associated with environmental stimuli such as amino acid- and insulin-related signals [36], we explored whether NaB also affected insulin- and amino acid-mediated activation of mTORC1. Our data showed that NaB could inhibit amino acid-induced mTORC1 activation, but it did not affect insulin-mediated activity. However, the activity of AKT, which is mainly controlled by insulin, was inhibited by NaB treatment. This may seem a bit paradoxical, but our recent study [60] and some previous studies have also found that amino acids can activate AKT [61–63]. However, whether the inhibition of AKT activity by butyrate is dependent on amino acid signaling remains to be further investigated. Moreover, AKT overexpression may exert a plethora of protective effects independent of mTOR stabilization, and we do not rule out that AKT may regulate ferroptosis through other mechanisms.

In conclusion, our work reveals the regulatory role of butyrate in ferroptosis and tumor growth. Our results reveal that the FFAR2-cAMP-PKA pathway is critical for GSK3-mediated degradation of NRF2, which plays an important role in the expression of SLC7A11 and cell ferroptosis. In addition, our work also reveals the regulatory role of the cAMP-PKA-mTORC1 pathway in NaB-induced ferroptosis by controlling the GPX4 protein synthesis. Functionally, our result showed the NaB treatment is correlated to the mTOR-dependent ferroptosis and development of colorectal cancer. Thus, this study offers new insights into the molecular mechanism of butyrate-induced ferroptosis that may be useful for identifying therapeutic targets for colorectal cancer treatment in the future.

## Supplementary information

Reproducibility checklist

Supplementary figure

Original Data File
