# Supplementary material for: Butyrate dictates ferroptosis sensitivity through FFAR2-mTOR signaling
Source: Cell Death Dis. 2023 Apr 25;14(4):292. doi: 10.1038/s41419-023-05778-0 (PMC10130170; doi:10.1038/s41419-023-05778-0)
Supplement: Supplementary file 2 — Supplementary figure [file 41419_2023_5778_MOESM2_ESM.pdf]

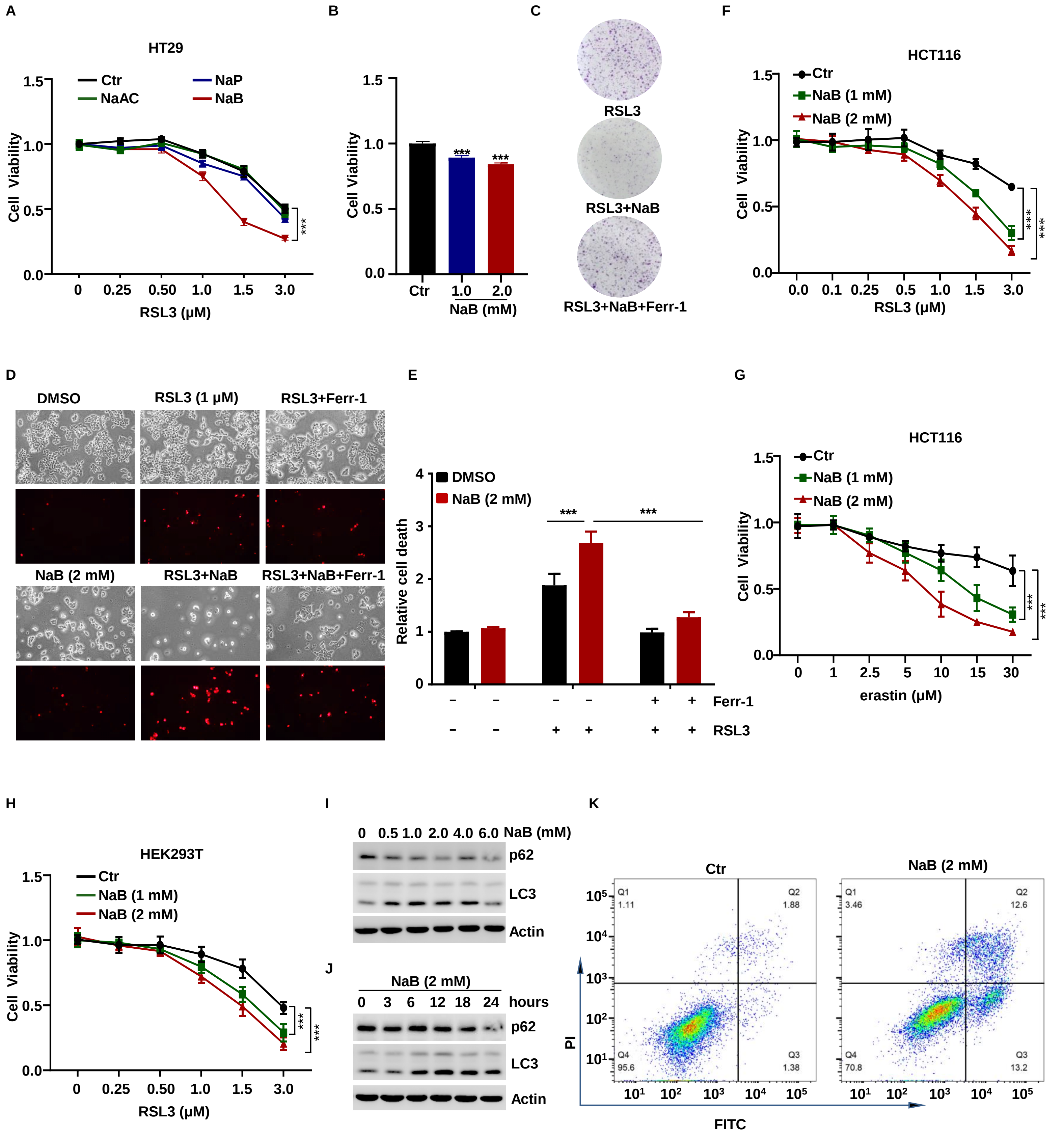

Fig. S1 Induction of cell death by butyrate in the presence of ferroptosis inducers

(A) HT29 cells were treated with different concentrations of RSL3 for 6 h in combination with indicated concentrations of NaAC (sodium acetate), NaP (sodium propionate), and NaB (sodium butyrate), and the viability of indicated cells was examined using CCK-8.

(B) HT29 cells were treated with different concentrations of NaB for 24 h, and the viability of cells were examined using CCK-8.

(C) HCT116 cells were treated with NaB (2 mM) for 24 h in combination with RSL3 (1 μM) or Ferr-1 (5 μM), then cell viability was detected by colony formation assay.

(D, E) HCT116 cells were treated with NaB (2 mM) for 24 h in combination with RSL3 (1 μM) or Ferr-1 (5 μM), then cell death was detected by dead cell staining assay (D) and the quantitative data is presented (E).

(F, G) HCT116 cells were treated with the indicated concentrations of RSL3 for 6 h (F) or erastin for 20 h (G) in combination with indicated concentrations of NaB, and the viability of cells were examined using CCK-8.

(H) HEK293T cells were treated with the indicated concentrations of RSL3 for 6 h in combination with indicated concentrations of NaB, and the viability of cells were examined using CCK-8.

(I) HT29 cells were treated with the indicated concentrations of NaB or in combination with bafilomycin A1 for 24 h, and autophagy was examined by measuring the levels of p62 and LC3II.

(J) HT29 cells were treated with NaB (2 mM) or in combination with bafilomycin A1 for indicated time, and autophagy was examined by measuring the levels of p62 and LC3II.

(K) HT29 cells were treated with NaB (2 mM) for 24 h, and apoptosis was examined using FACS.

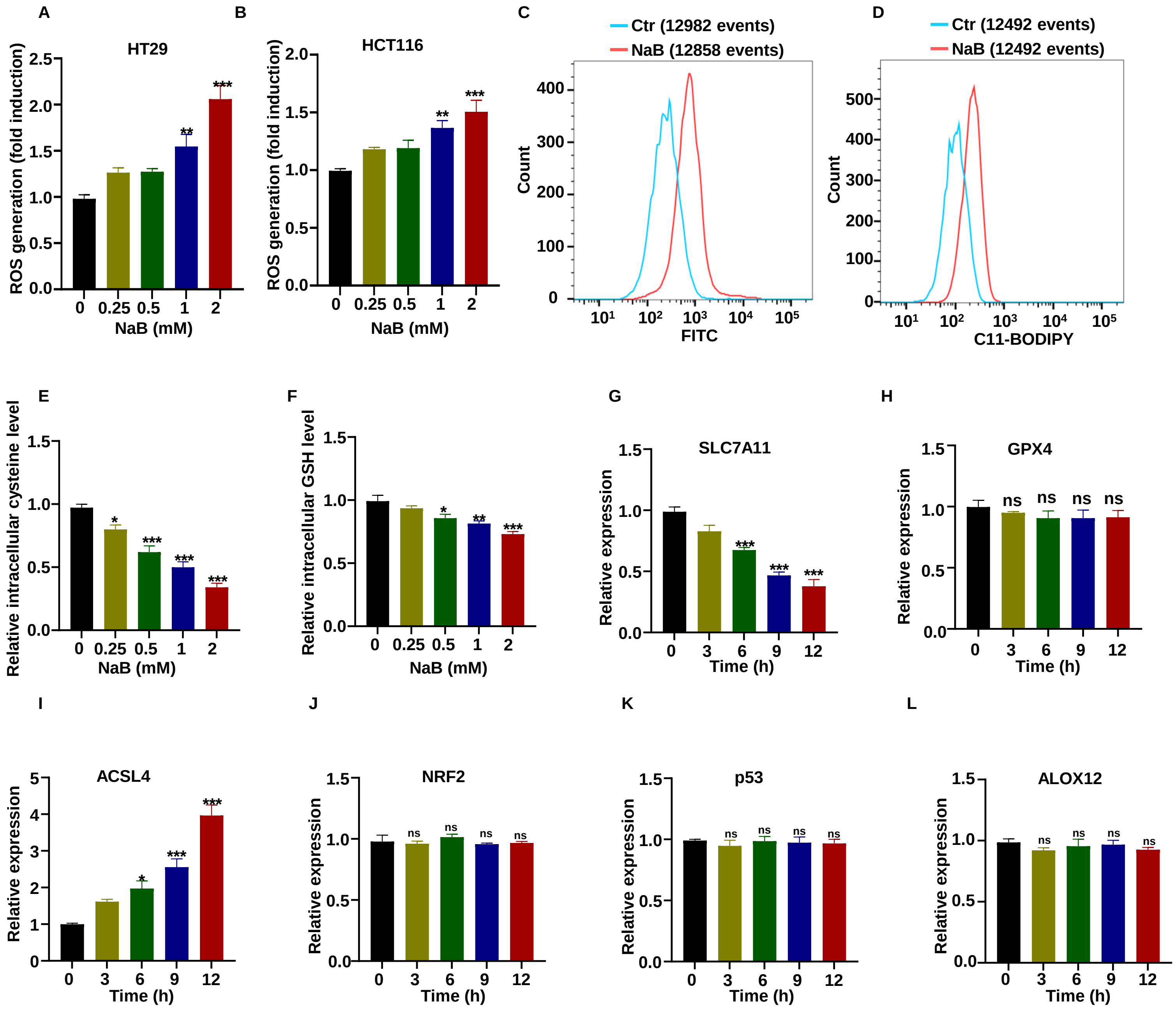

Fig. S2 Promotion of ferroptosis by butyrate via increase in lipid ROS production and decrease in the expression of SLC7A11

(A, B) HT29 (A), HCT116 (B) were treated with different concentrations of NaB (2 mM) for 24 h, and ROS production was determined using a microplate-reader. For statistical analysis, fluorescence intensity was normalized against the control and expressed as fold-change.

(C) HCT116 cells were treated with NaB (2 mM) for 24 h, ROS levels were measured using flow cytometer. For statistical analysis, fluorescence intensity was normalized against the control and expressed as fold-change.

(D) HCT116 cells were treated with NaB (2 mM) for 24 h. Lipid ROS levels were measured using a flow cytometer. For statistical analysis, fluorescence intensity was normalized against the control and expressed as fold-change.

(E, F) HCT116 were treated with different concentrations of NaB for 6 h, and relative cysteine (E) and GSH (F) levels were showed. All samples were normalized to cell number and conducted with three independent replicates.

(G-L) HCT116 cells were treated with NaB (2 mM) for the indicated time, and the expression of SLC7A11 (G), GPX4 (H), ACSL4 (I), NRF2 (J), p53 (K), and ALOX12 (L) were measured using qRT-PCR.

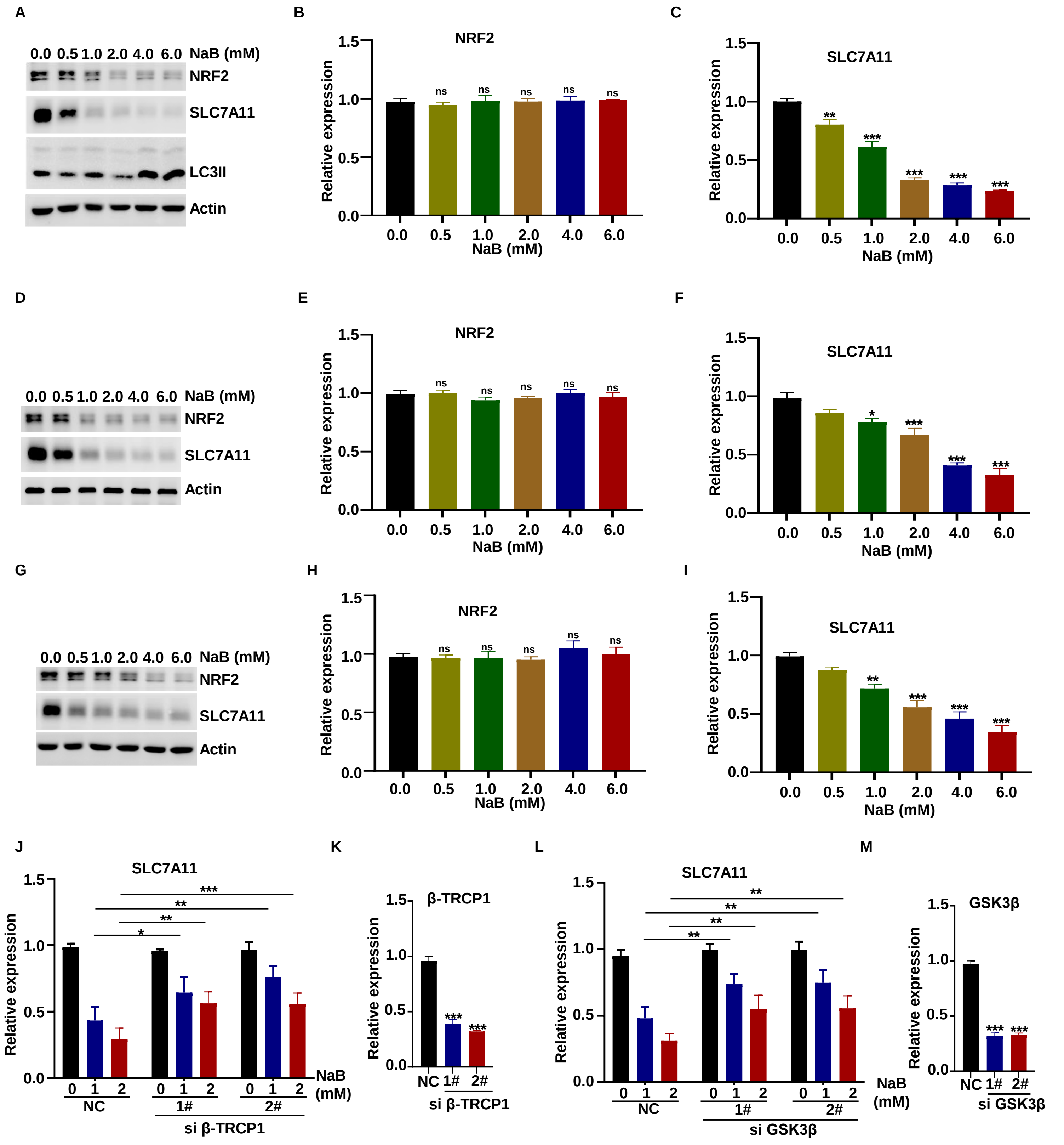

Fig. S3 Butyrate-induced SLC7A11 downregulation via the GSK3β-β-TRCP1-NRF2 pathway

(A-C) HCT116 cells were treated with the indicated concentrations of NaB for 6 h, the levels of the indicated proteins were evaluated by western blot (A), the expression of NRF2 (B) and SLC7A11 (C) were measured using qRT-PCR.

(D-F) Caco2 cells were treated with the indicated concentrations of NaB for 6 h, the levels of the indicated proteins were evaluated by western blot (D), the expression of NRF2 (E) and SLC7A11 (F) were measured using qRT-PCR.

(G-I) MC38 cells were treated with the indicated concentrations of NaB for 6 h, the levels of the indicated proteins were evaluated by western blot (G), the expression of NRF2 (H) and SLC7A11 (I) were measured using qRT-PCR.

(J-M) β-TRCP1-(J, K) or GSK3β-knockdown (L, M) HT29 cells were treated with the indicated concentrations of NaB for 6 h, and the expression of SLC7A11, β-TRCP1, and GSK3β were measured using qRT-PCR. The knockdown efficiency were shown in (K) and (M).

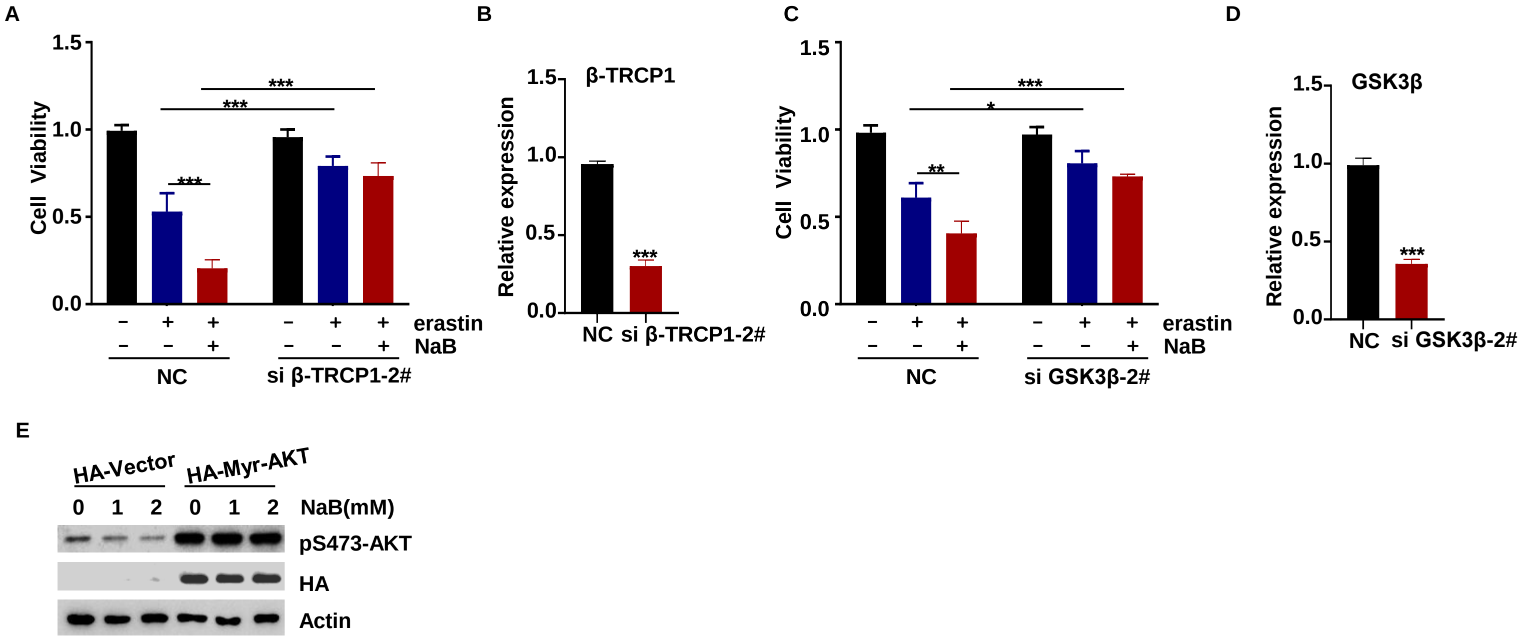

Fig. S4 Promotion of ferroptosis by butyrate via the mTORC2-AKT-GSK3β pathway
(A-D). β-TRCP1-(A, B) or GSK3β-knockdown (C, D) HT29 cells were treated with erastin (10 μM) for 20 h in combination with NaB (2 mM), and the viability of indicated cells were examined using CCK-8. The expression of β-TRCP1 and GSK3β were measured using qRT-PCR. The knockdown efficiency were shown in (B) and (D). (E) Myr-AKT overexpressed HCT116 cells were treated with different concentrations of NaB for 6 h, the levels of pS473-AKT, HA and the indicated proteins were evaluated by western blot.

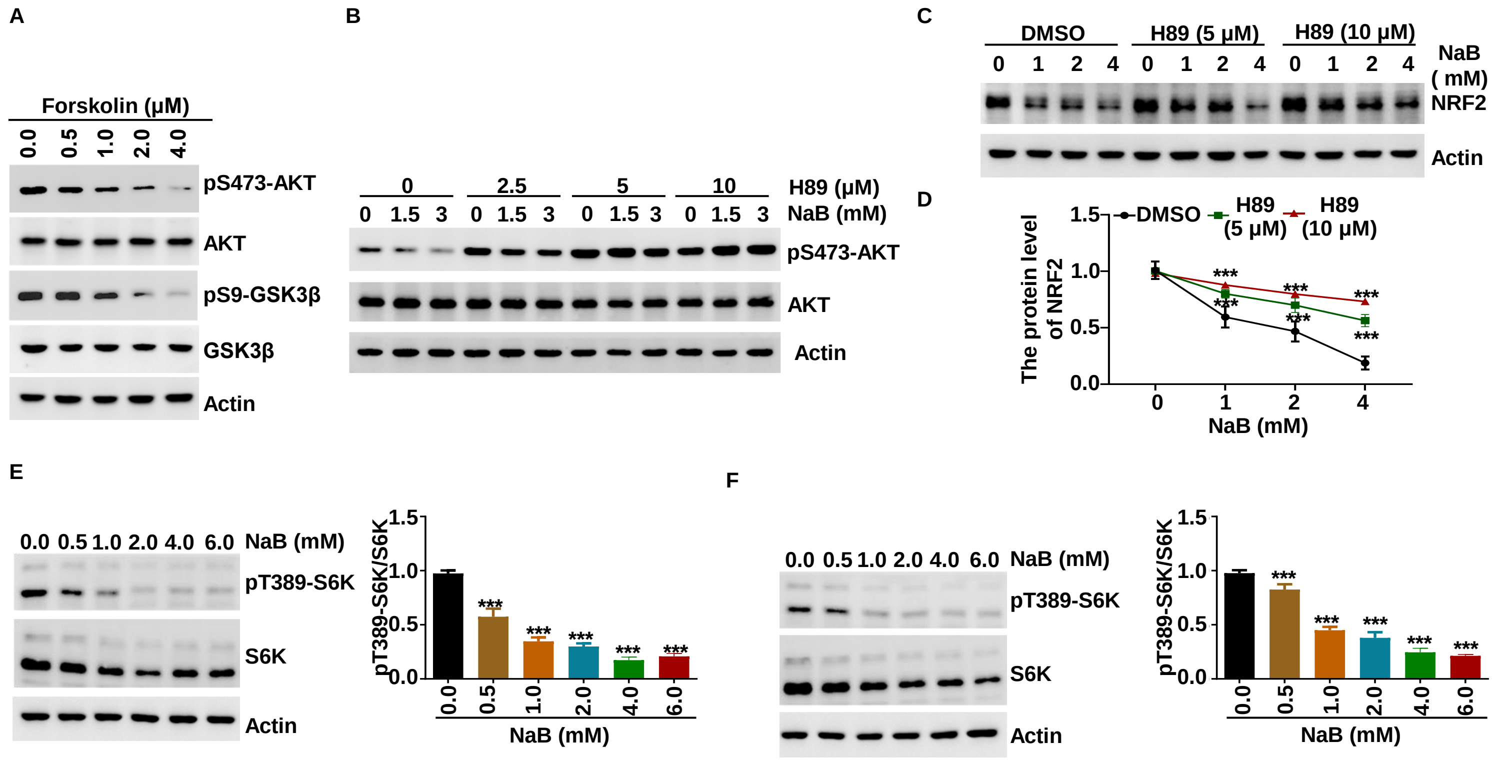

Fig. S5 Promotion of ferroptosis by butyrate via cAMP-PKA-mediated AKT-GSK3 activation
(A) HT29 cells were treated with the indicated concentrations of Forskolin for 6 h, the levels of pS473-AKT, p-GSK3β and the indicated proteins were evaluated by western blot.
(B) HT29 cells were treated with the indicated concentrations of NaB or in combination with H89 for 6 h, the levels of pS473-AKT and indicated proteins were evaluated by western blot.
(C, D) HT29 cells were treated with H89 or in combination with the indicated concentration NaB for 6 h, and then western blot was used to evaluate the levels of NRF2 (C), quantitative data for NRF2 protein levels are presented (D).
(E) HCT116 cells were treated with the indicated concentrations of NaB for 6 h, and then western blot was used to evaluate the levels of pT389-S6K.
(F) Caco2 cells were treated with the indicated concentrations of NaB for 6 h, and then western blot was used to evaluate the levels of pT389-S6K.
