# Supplementary material for: Butyrate dictates ferroptosis sensitivity through FFAR2-mTOR signaling
Source: Cell Death Dis. 2023 Apr 25;14(4):292. doi: 10.1038/s41419-023-05778-0 (PMC10130170; doi:10.1038/s41419-023-05778-0)
Supplement: Supplementary file 3 — Original Data File [file 41419_2023_5778_MOESM3_ESM.pdf]

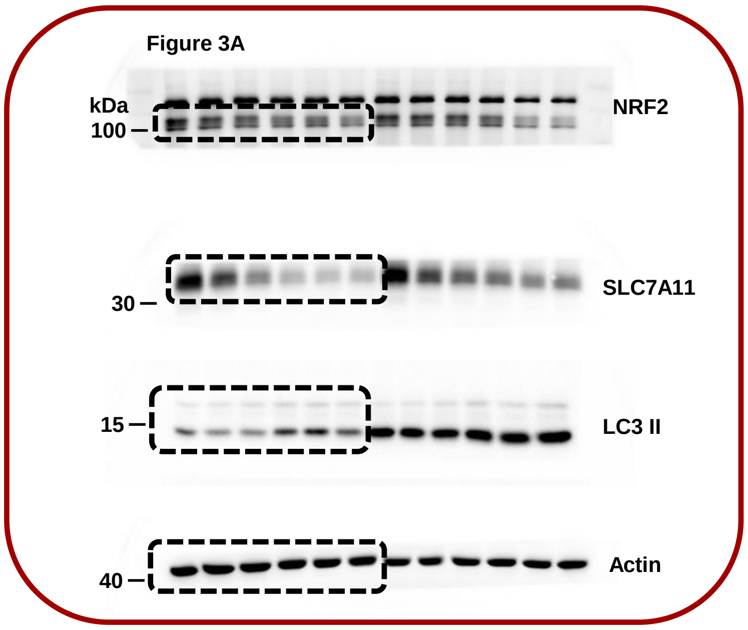
Figure 3A
kDa
100
NRF2
30
SLC7A11
15
LC3 II
40
Actin

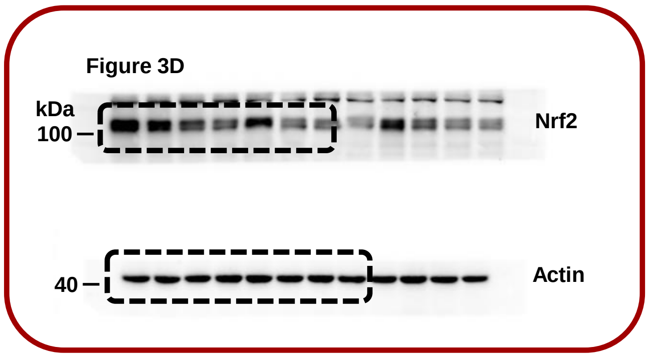
Figure 3D
kDa
100
Nrf2
40
Actin

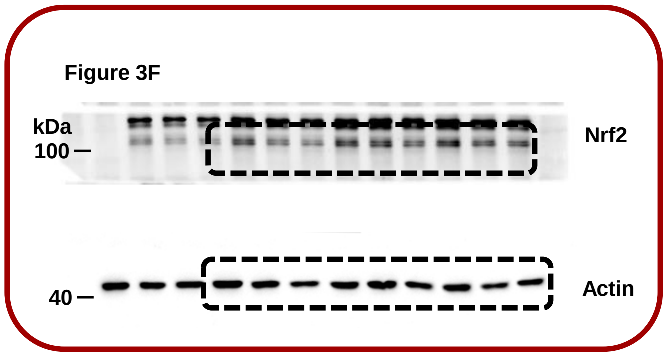
Figure 3F
kDa
100
Nrf2
40
Actin

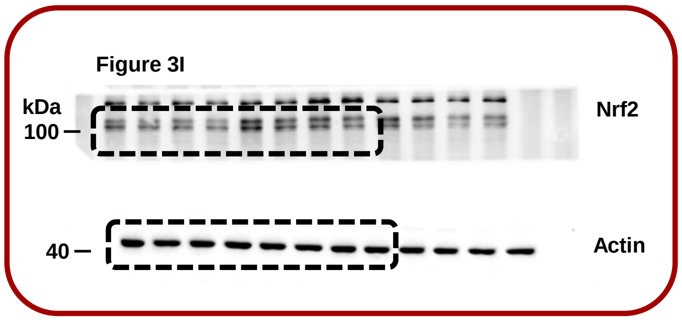
Figure 3I
kDa
100
Nrf2
40
Actin

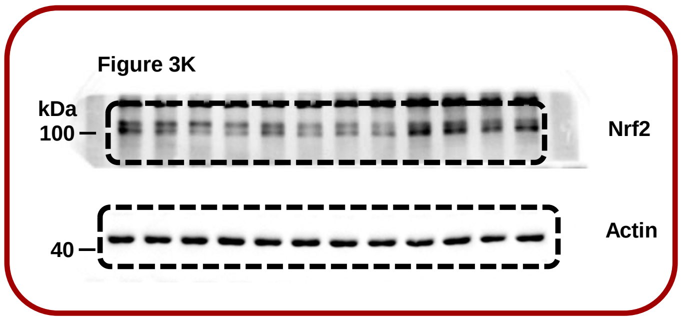
Figure 3K
kDa
100
Nrf2
40
Actin

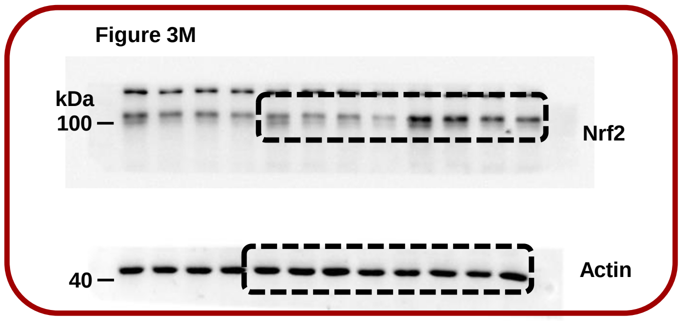
Figure 3M
kDa
100
Nrf2
40
Actin

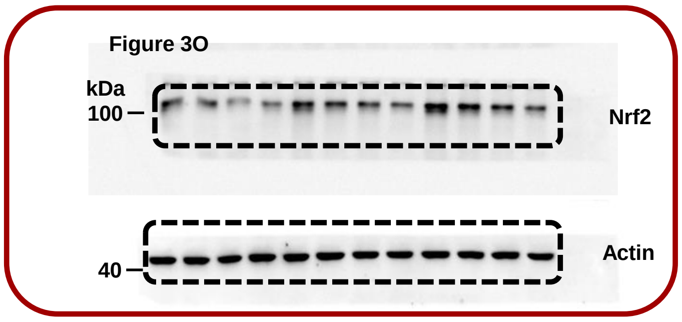
Figure 3O
kDa
100
Nrf2
40
Actin

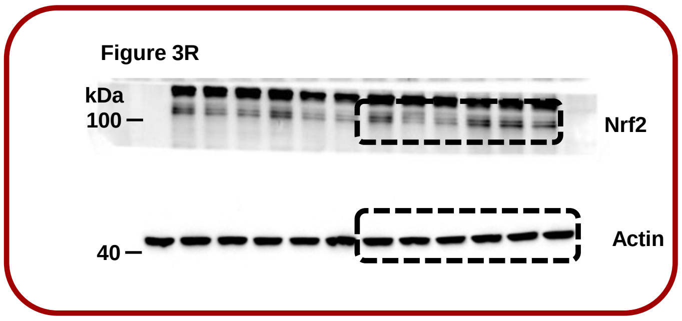
Figure 3R
kDa
100
Nrf2
40
Actin

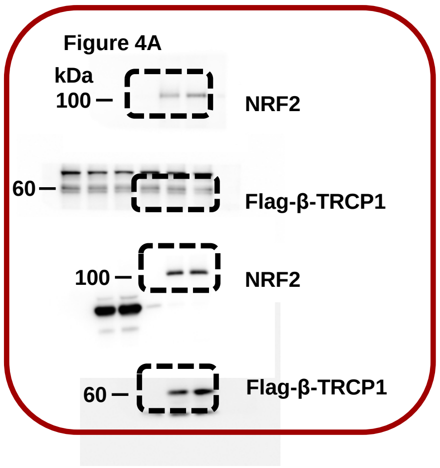
Figure 4A
kDa
100
NRF2
60
Flag-β-TRCP1
100
NRF2
60
Flag-β-TRCP1

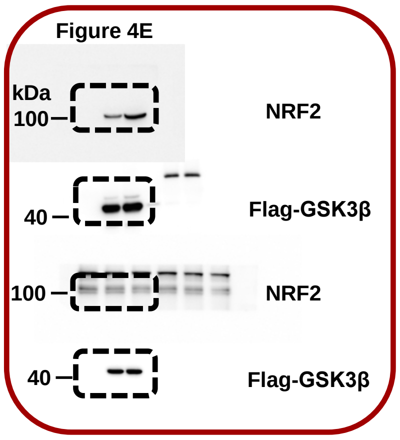
Figure 4E
kDa
100
NRF2
40
Flag-GSK3β
100
NRF2
40
Flag-GSK3β

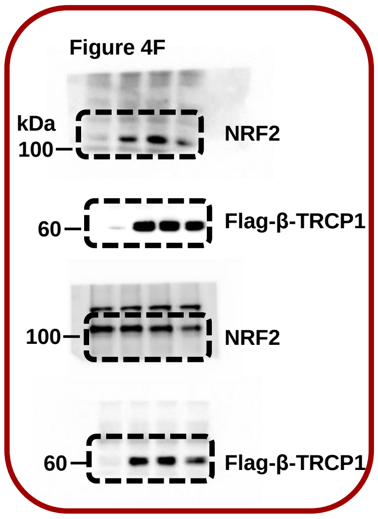
Figure 4F
kDa
100
NRF2
60
Flag-β-TRCP1
100
NRF2
60
Flag-β-TRCP1

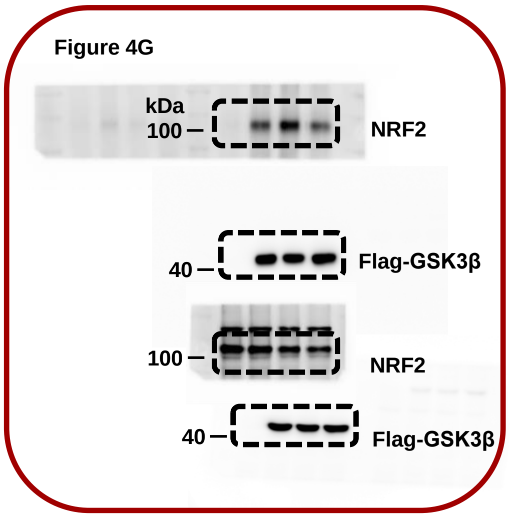
Figure 4G
kDa
100
NRF2
40
Flag-GSK3β
100
NRF2
40
Flag-GSK3β

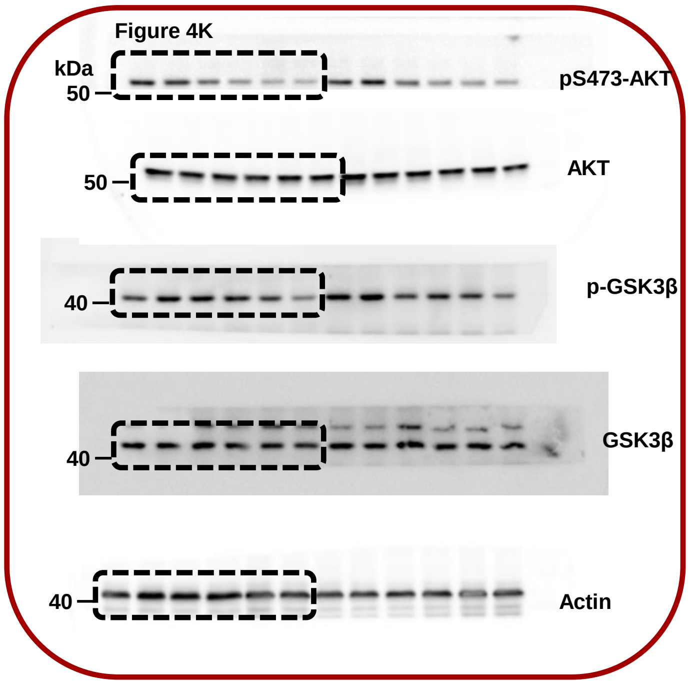
Figure 4K
kDa
50
pS473-AKT
50
AKT
40
p-GSK3β
40
GSK3β
40
Actin

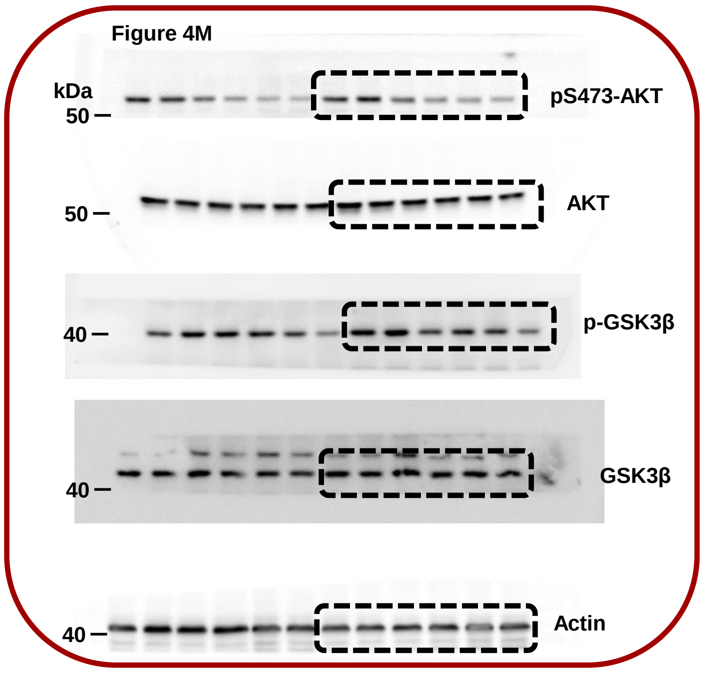
Figure 4M
kDa
50
pS473-AKT
50
AKT
40
p-GSK3β
40
GSK3β
40
Actin

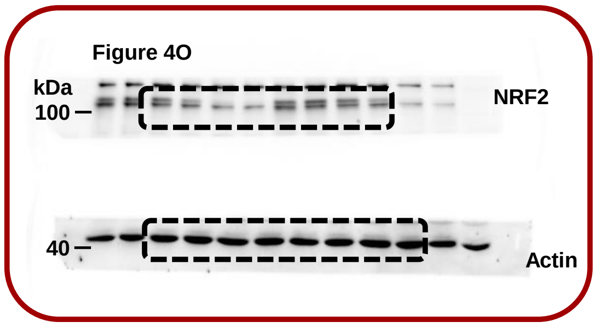
Figure 4O
kDa
100
NRF2
40
Actin

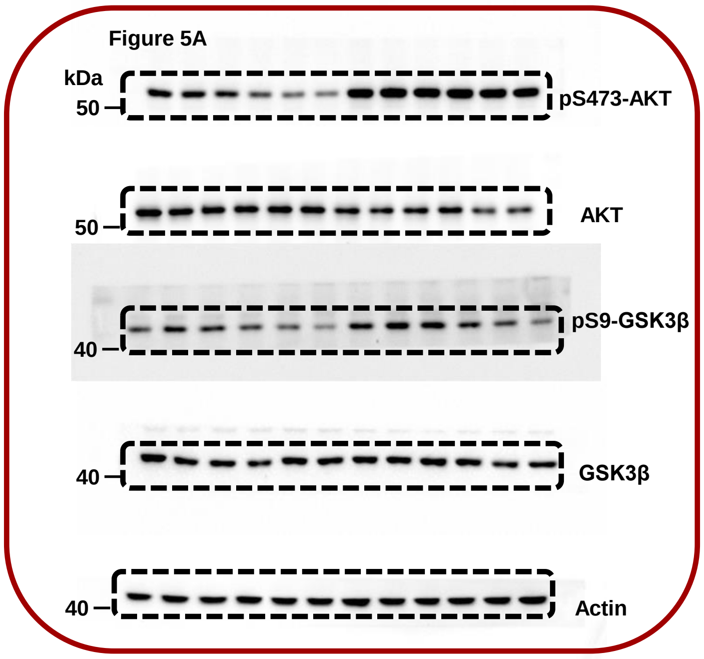
Figure 5A
kDa
50
pS473-AKT
50
AKT
40
pS9-GSK3β
40
GSK3β
40
Actin

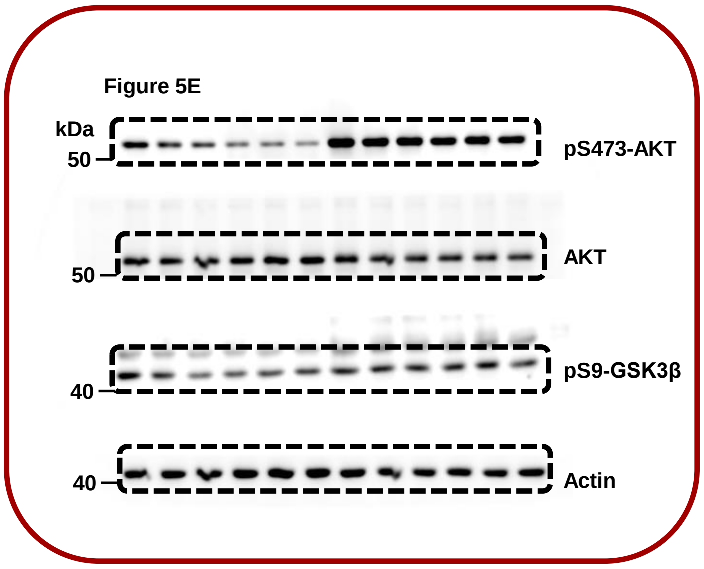
Figure 5E
kDa
50
pS473-AKT
50
AKT
40
pS9-GSK3β
40
Actin

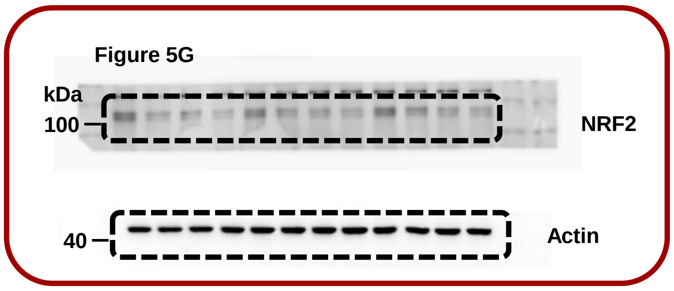
Figure 5G
kDa
100
NRF2
40
Actin

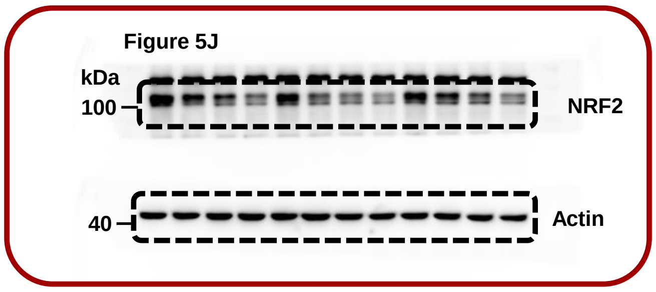
Figure 5J
kDa
100
NRF2
40
Actin

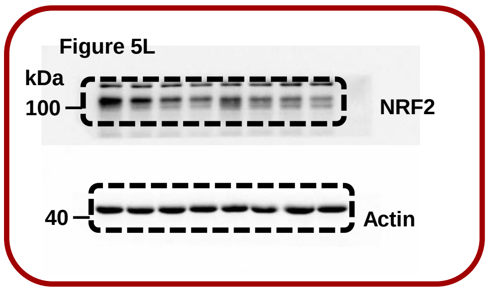
Figure 5L
kDa
100
NRF2
40
Actin

Figure 6B

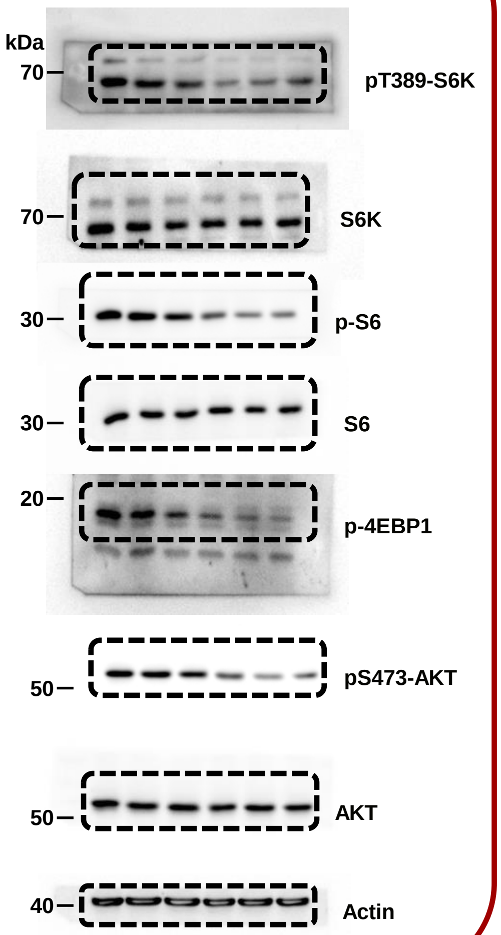

Figure 6D

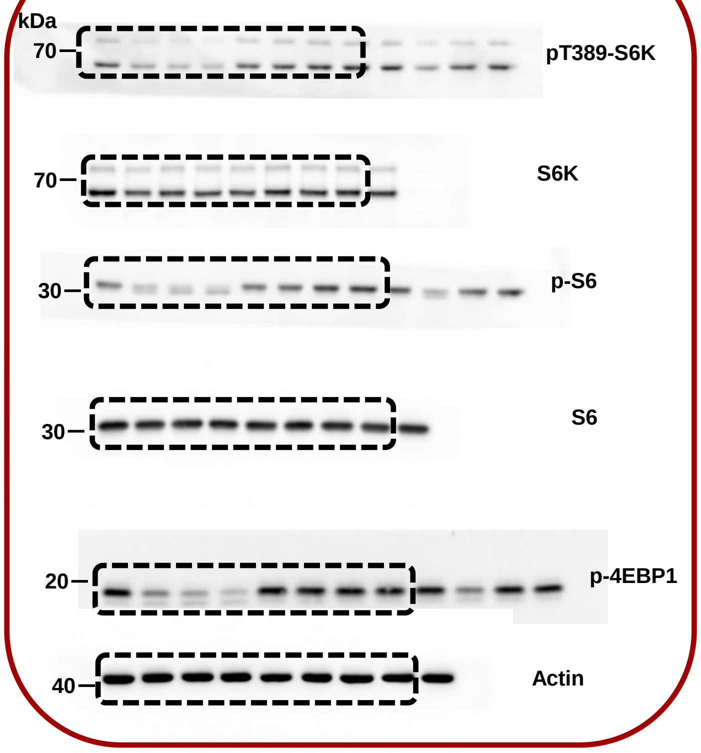

Figure 6G

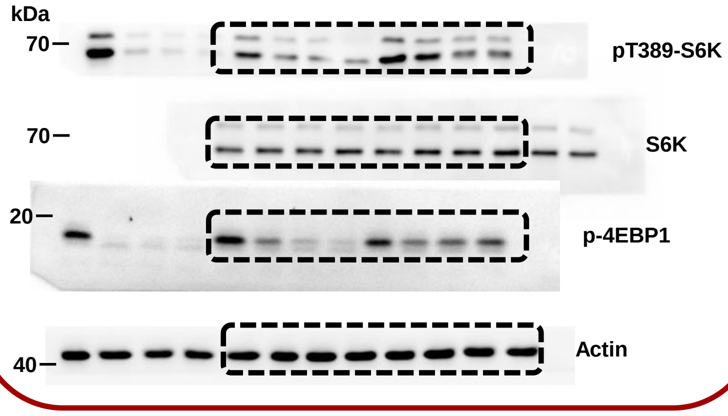

Figure 6I

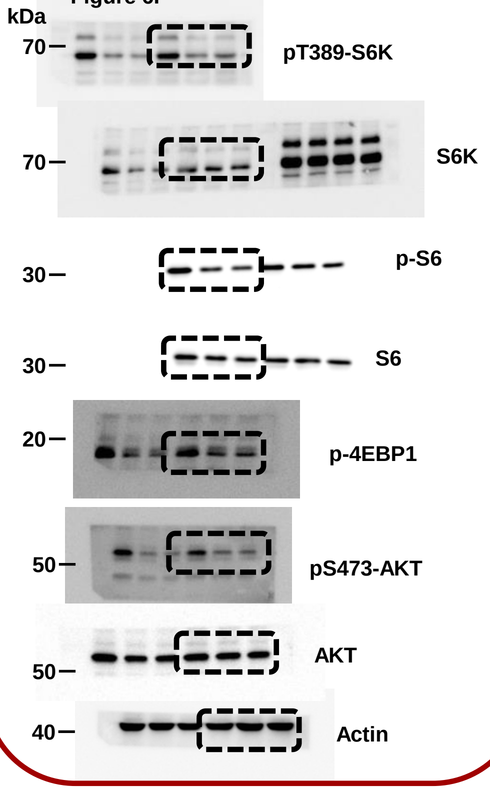

Figure 6K

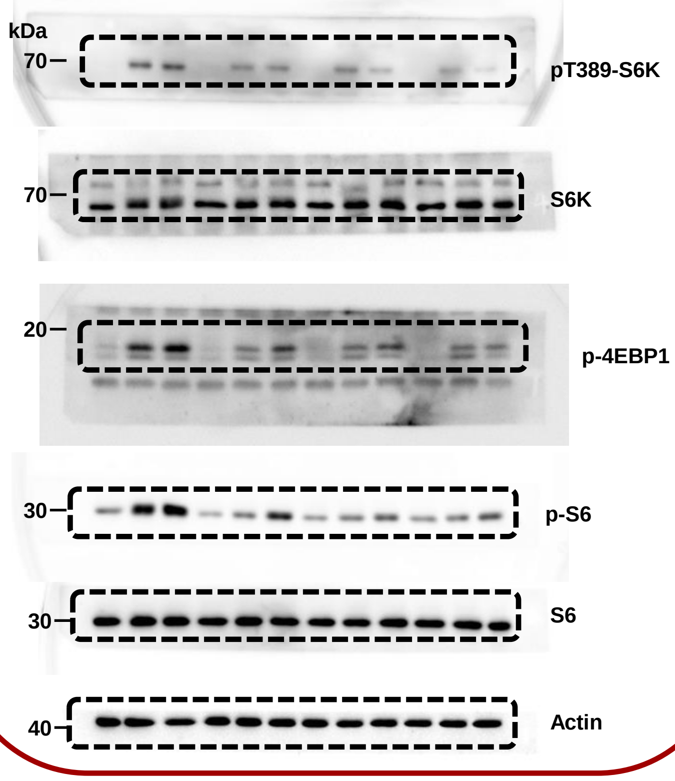

Figure 6M

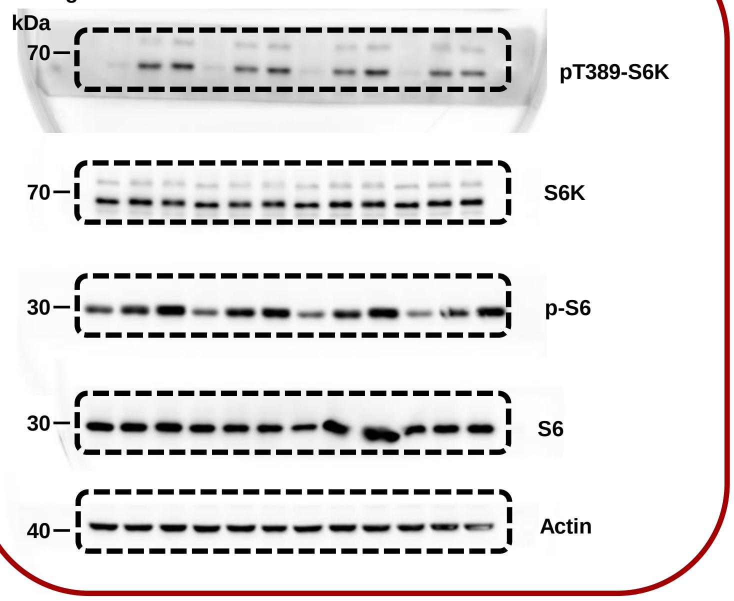

Figure 6O

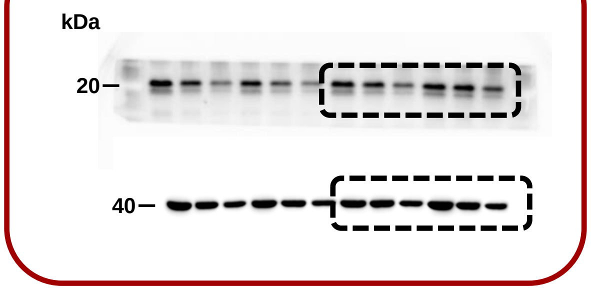

Figure 6R

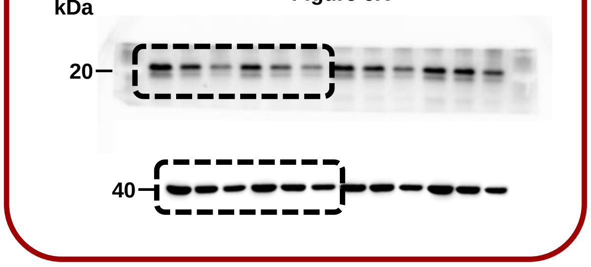

Figure 7E

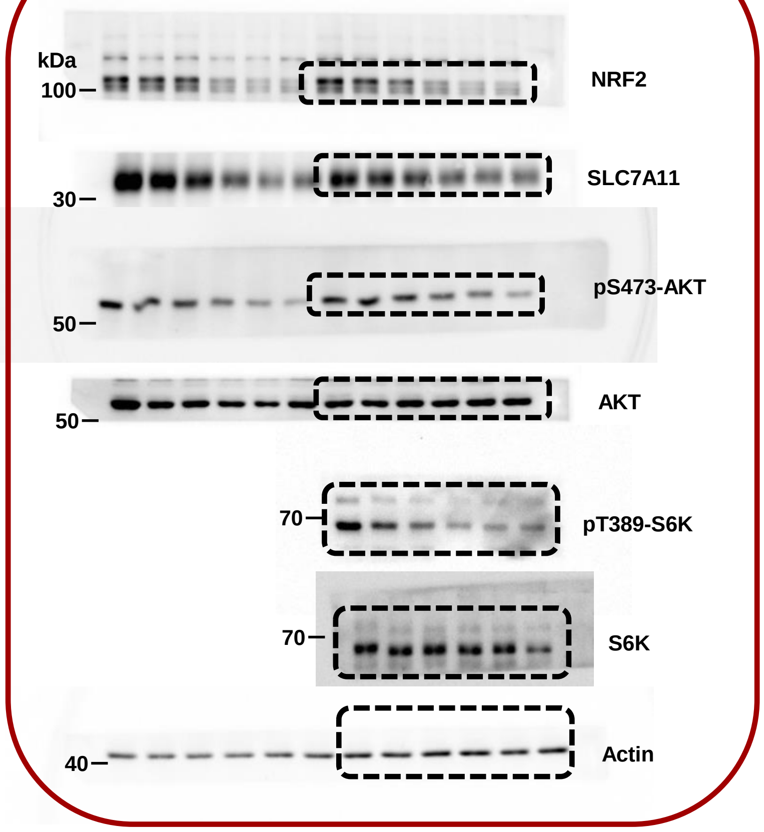

Figure 7M

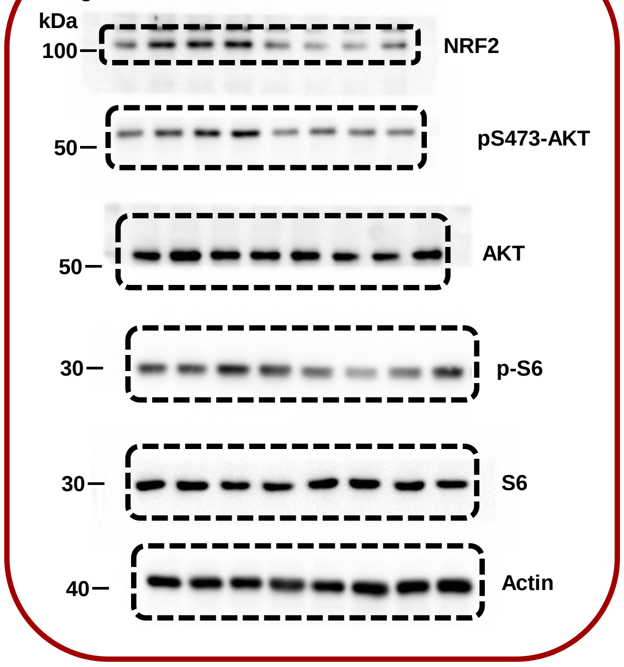

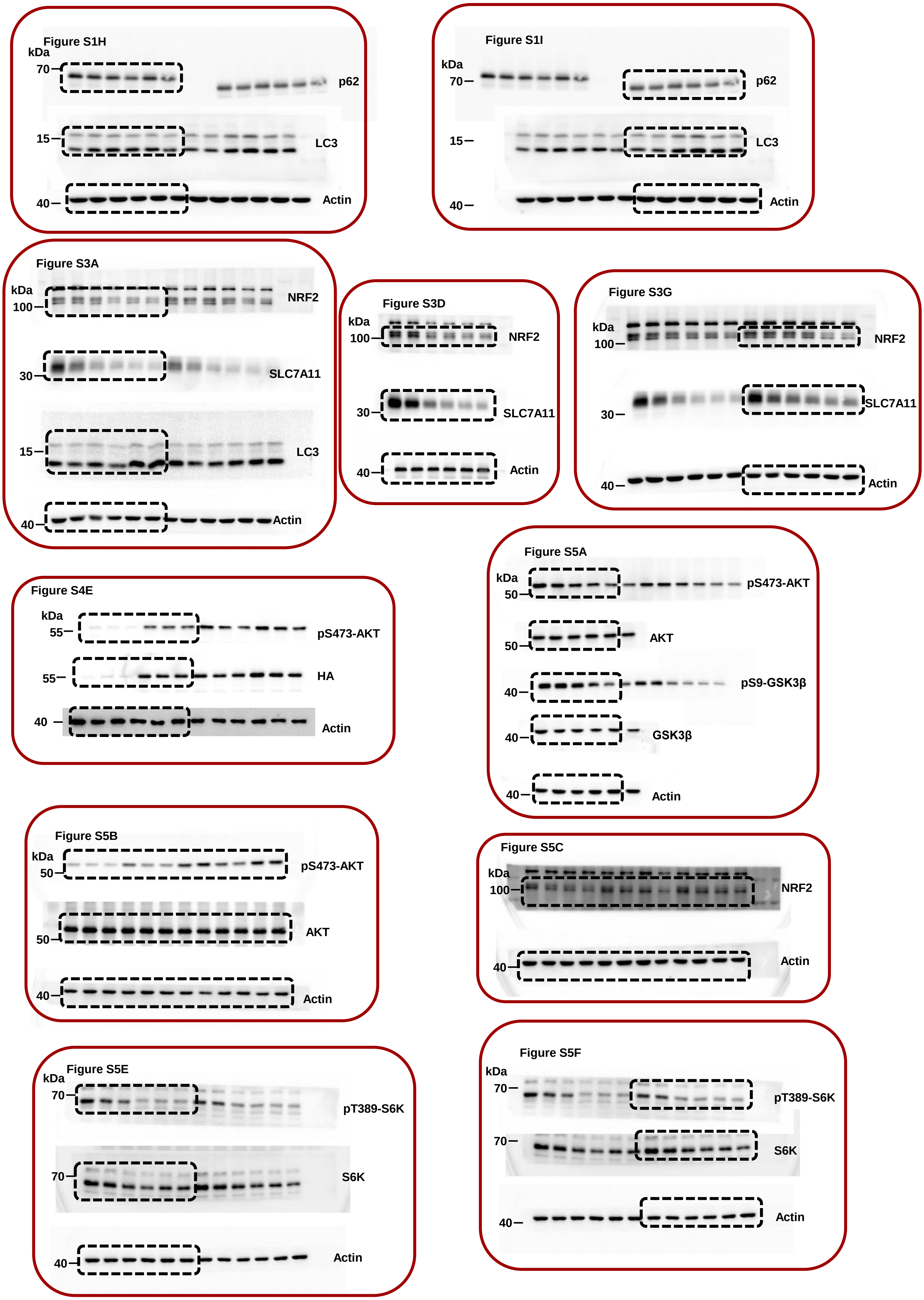
Figure S1H
kDa
70
p62
15
LC3
40
Actin
Figure S1I
kDa
70
p62
15
LC3
40
Actin
Figure S3A
kDa
100
NRF2
30
SLC7A11
15
LC3
40
Actin
Figure S3D
kDa
100
NRF2
30
SLC7A11
40
Actin
Figure S3G
kDa
100
NRF2
30
SLC7A11
40
Actin
Figure S4E
kDa
55
pS473-AKT
55
HA
40
Actin
Figure S5A
kDa
50
pS473-AKT
50
AKT
40
pS9-GSK3β
40
GSK3β
40
Actin
Figure S5B
kDa
50
pS473-AKT
50
AKT
40
Actin
Figure S5C
kDa
100
NRF2
40
Actin
Figure S5E
kDa
70
pT389-S6K
70
S6K
40
Actin
Figure S5F
kDa
70
pT389-S6K
70
S6K
40
Actin
